# Genetic depletion of the RNA helicase DDX3 leads to impaired elongation of translating ribosomes triggering co-translational quality control of newly synthesized polypeptides

**DOI:** 10.1093/nar/gkab667

**Published:** 2021-08-06

**Authors:** Prasad Kottayil Padmanabhan, Gabriel Reis Ferreira, Ouafa Zghidi-Abouzid, Camila Oliveira, Carole Dumas, Filipe Colaço Mariz, Barbara Papadopoulou

**Affiliations:** Research Center in Infectious Diseases, Division of Infectious Disease and Immunity CHU de Quebec Research Center-University Laval, Quebec, QC G1V 4G2, Canada; Department of Microbiology, Infectious Disease and Immunology, Faculty of Medicine, University Laval, Quebec, QC G1V 4G2, Canada; Research Center in Infectious Diseases, Division of Infectious Disease and Immunity CHU de Quebec Research Center-University Laval, Quebec, QC G1V 4G2, Canada; Department of Microbiology, Infectious Disease and Immunology, Faculty of Medicine, University Laval, Quebec, QC G1V 4G2, Canada; Research Center in Infectious Diseases, Division of Infectious Disease and Immunity CHU de Quebec Research Center-University Laval, Quebec, QC G1V 4G2, Canada; Department of Microbiology, Infectious Disease and Immunology, Faculty of Medicine, University Laval, Quebec, QC G1V 4G2, Canada; Research Center in Infectious Diseases, Division of Infectious Disease and Immunity CHU de Quebec Research Center-University Laval, Quebec, QC G1V 4G2, Canada; Department of Microbiology, Infectious Disease and Immunology, Faculty of Medicine, University Laval, Quebec, QC G1V 4G2, Canada; Research Center in Infectious Diseases, Division of Infectious Disease and Immunity CHU de Quebec Research Center-University Laval, Quebec, QC G1V 4G2, Canada; Department of Microbiology, Infectious Disease and Immunology, Faculty of Medicine, University Laval, Quebec, QC G1V 4G2, Canada; Research Center in Infectious Diseases, Division of Infectious Disease and Immunity CHU de Quebec Research Center-University Laval, Quebec, QC G1V 4G2, Canada; Department of Microbiology, Infectious Disease and Immunology, Faculty of Medicine, University Laval, Quebec, QC G1V 4G2, Canada; Research Center in Infectious Diseases, Division of Infectious Disease and Immunity CHU de Quebec Research Center-University Laval, Quebec, QC G1V 4G2, Canada; Department of Microbiology, Infectious Disease and Immunology, Faculty of Medicine, University Laval, Quebec, QC G1V 4G2, Canada

## Abstract

DDX3 is a multifaceted RNA helicase of the DEAD-box family that plays central roles in all aspects of RNA metabolism including translation initiation. Here, we provide evidence that the *Leishmania* DDX3 ortholog functions in post-initiation steps of translation. We show that genetic depletion of DDX3 slows down ribosome movement resulting in elongation-stalled ribosomes, impaired translation elongation and decreased *de novo* protein synthesis. We also demonstrate that the essential ribosome recycling factor Rli1/ABCE1 and termination factors eRF3 and GTPBP1 are less recruited to ribosomes upon DDX3 loss, suggesting that arrested ribosomes may be inefficiently dissociated and recycled. Furthermore, we show that prolonged ribosome stalling triggers co-translational ubiquitination of nascent polypeptide chains and a higher recruitment of E3 ubiquitin ligases and proteasome components to ribosomes of DDX3 knockout cells, which further supports that ribosomes are not elongating optimally. Impaired elongation of translating ribosomes also results in the accumulation of cytoplasmic protein aggregates, which implies that defects in translation overwhelm the normal quality controls. The partial recovery of translation by overexpressing Hsp70 supports this possibility. Collectively, these results suggest an important novel contribution of DDX3 to optimal elongation of translating ribosomes by preventing prolonged translation stalls and stimulating recycling of arrested ribosomes.

## INTRODUCTION

Translation is a fundamental biological process that decodes genetic information into functional proteins. The process of translation is divided into four functionally distinct phases: initiation, elongation, termination and ribosome recycling. Initiation has traditionally been considered to be rate-limiting and thus the focus of regulation (1). However, increasing evidence in different organisms supports that translation elongation can also be regulatory and central to determining protein fate and to control development and cellular stress (2). We now know that translation elongation is not uniform across the length of the mRNA, with the ribosome accelerating and slowing down during elongation (3). The elongation phase of translation can fail for a variety of reasons, resulting in stalled ribosomes. There are many causes that can trigger ribosome stalling including aberrant or highly structured mRNAs, translation of 3′UTRs, decoding of suboptimal codons, changes in tRNA levels, misfolded polypeptides, and defective ribosomes (4,5). Stalling of ribosomes can generate non-functional and potentially harmful polypeptides. Thus, all cells have evolved specialized ribosome-associated quality control (RQC) pathways to sense truncated protein products generated by ribosomes that stall on aberrant/defective mRNAs and eliminate the non-functional nascent peptides through ubiquitylation and transfer to the 26S proteasome for degradation (4,6,7).

The DEAD-box family of RNA helicases defined by the Asp-Glu-Ala-Asp (DEAD) motif is composed of ubiquitous proteins belonging to the largest family of superfamily 2 helicases that are found in nearly all organisms and are involved in virtually all processes of RNA metabolism and regulation in the cell (8,9). Through their linked activities of ATP-dependent RNA-binding proteins and RNA-dependent ATPases they are able to unwind RNA–RNA duplexes, alter RNA–protein interactions, remodel ribonucleoprotein complexes, and act as RNA chaperones (8,9). All DEAD-box RNA helicases share a conserved core that consists of two RecA-like domains connected via a short flexible linker that allows changes in their orientation, which is critical for the enzyme functions (8). RecA-like domains are composed of twelve conservative motifs that function in ATP binding or hydrolysis, RNA binding and RNA strands unwinding. The core is flanked by variable subfamily-specific N- and C-terminal extensions, which allow interaction with other proteins or RNA (8,9).

The Ded1/DDX3 subfamily of DEAD-box helicases are of particular interest as members function during protein translation, are essential for viability, and are frequently altered in human malignancies and in the context of several viral infections (10). The human genome encodes two types of *DDX3* genes, *DDX3X* and its homolog *DDX3Y* that are members of the Ded1/DDX3 subfamily, along with the *Saccharomyces cerevisiae* ortholog Ded1p, Xenopus An3, mouse PL10 and *Drosophila* Belle (11). Human DDX3 is a component of several messenger ribonucleoproteins that are found in the translation initiation machineries (12–14). DDX3 has also been implicated in the translational response to stress (15,16). Similarly, Ded1p has been linked primarily to translation initiation either through specific interactions with key initiation factors (17–19) or by promoting the assembly of the 48S pre-initiation complex (20) or by resolving secondary structures particularly in 5′UTRs (18,20,21). The *Drosophila* Belle ortholog has equally been shown to regulate translation of specific sets of germline transcripts (22).

Virtually all sequenced eukaryotes encode one or more highly similar DDX3X orthologs (11). The *Leishmania* genome encodes a single DDX3 homolog (LINF_320009100; 614 aa) which shares 54%, 53% and 52% sequence identity with DDX3X, DDX3Y and Ded1p, respectively (23). As a reference, DDX3X shares 51% sequence identity to its *Saccharomyces cerevisiae* Ded1p ortholog (11). In addition to the twelve signature motifs found in the ‘helicase core’ of Ded1/DDX3 subfamily members, DDX3 orthologs have specific N- and C-terminal unstructured regions of low sequence complexity that harbor few conserved motifs of not yet well-defined functions (11). Interestingly, the *Leishmania* DDX3-like protein harbors the N-terminal extension motif CINF and the C-terminal extension RDYR motif (RGGYR in the *Leishmania* homolog), which is important for RNA duplex unwinding and also for protein oligomerization (24) (see Supplementary Figure S1). Recently, an extensive BLAST and phylogenetic analysis of potential members of the Ded1/DDX3 subfamily in *Leishmania infantum* similarly concluded that LINF_320009100 was the most likely ortholog of the Ded1/DDX3 subfamily in *Leishmania* and related trypanosomes (25). The closely related DDX3 paralog in *Leishmania*, LINF_350036300 (Dbp1; 924 aa) belongs to the related *Drosophila* Vasa/DDX4 subfamily (25) and it was shown previously in *L. amazonensis* to be expressed predominantly in the invertebrate promastigote stage of the parasite (26). DDX3-like genes are generally essential in the organisms tested. Genetic inactivation of the single copy *DDX3* gene in *L. major* and *L. infantum* promastigotes grown under ‘unstressed’ conditions resulted in viable albeit slow-growing parasites (23). However, DDX3 knockout parasites were unable to survive under heat or oxidative stress and experienced proteotoxic stress (23). Moreover, DDX3 was essential for survival of amastigote forms encountering a variety of stress stimuli inside their mammalian macrophage host that alter the parasite's translation (23). The essential function of DDX3 in *Leishmania* amastigotes (23) is in line with previous studies indicating higher expression of this protein in this parasite life stage (26). Of relevance to translation regulation, we have shown previously that the *Leishmania* DDX3-like protein protects ribosomal RNA from degradation under conditions of stress and drug-induced cell death (27). DEAD-box RNA helicases are poorly studied in parasitic protozoa and their role in translation regulation is yet to be established.

In this study, we provide several lines of evidence supporting that the *Leishmania* DDX3 ortholog functions in post-initiation steps of translation. We show that ribosome movement slows down in cells lacking DDX3 resulting in elongation-stalled ribosomes, reduced translation elongation, and impaired protein synthesis. We further demonstrate that the essential ribosome recycling factor Rli1/ABCE1 and the translational GTPases eRF3 and GTPBP1 known to regulate termination/recycling and mRNA surveillance are less recruited to the ribosome in the absence of DDX3, which suggests that arrested ribosomes may be inefficiently dissociated and recycled. In addition, we show that perturbed translation elongation upon DDX3 loss leads to increased co-translational ubiquitination of nascent polypeptides. In line with increased co-translational ubiquitination we show that E3 ubiquitin ligases and proteasome components are recruited at much higher levels to the ribosomes. Impaired elongation of translating ribosomes in DDX3 knockout cells results in the accumulation of cytoplasmic protein aggregates, suggesting that defects in protein synthesis overwhelm the normal quality controls. Altogether, these data highlight a novel and important contribution of the *Leishmania* DDX3 ortholog to the optimal elongation of translating ribosomes by preventing prolonged translation stalls and stimulating dissociation and recycling of arrested ribosomes.

## MATERIALS AND METHODS

### Strains and cell culture

The parental strain *Leishmania infantum* MHOM/MA/67/ITMAP-263 was used in this study. The *L. infantum* DDX3 (LINF_320009100, http://tritrypdb.org) knockout strain (*Li*DDX3^(−/−)^) and the add-back mutant (*Li*DDX3^(−/−)^REV) ectopically expressing a DDX3 copy tagged at the C-terminus with an HA epitope (DDX3-HA) have been described previously (27). *L. infantum* promastigotes were cultured at pH 7.0 and 25°C in SDM-79 medium supplemented with 10% heat-inactivated fetal calf serum (FCS) (Multicell Wisent Inc., Canada) and 5 μg/ml hemin. The susceptibility of the above strains to translation inhibitors was assessed by growing *L. infantum* promastigotes in the presence of various concentrations of cycloheximide (Sigma) (0–400 ng/ml), or puromycin dihydrochloride (Thermo Fisher Scientific) (0–25 μg/ml).

### Plasmid constructs and transfections

The deletion mutants *Li*DDX3ΔDEADRM and *Li*DDX3ΔSAT have been reported previously (23). To engineer point mutations P219S, F393L and GG to AA in the *L. infantum* DDX3 protein, the Phusion DNA polymerase (NEB)-based PCR strategy was used (see Supplementary Table S1 for primers). The HA-tagged DDX3 mutant proteins were cloned into the XbaI and HindIII sites of the zeocin (ZEO)-expressing vector pSPαIR-ZEO-αIR (28). The genes encoding the 60S subunit ribosomal protein L13a (LINF_150007100), the 40S ribosomal protein S6 (LINF_150022800), and the eukaryotic release factor 3 (eRF3) (LINF_110017700) were PCR-amplified from *L. infantum* genomic DNA using specific primers to include an HA epitope-tag at the C-terminus (Supplementary Table S1), then cloned into the XbaI and HindIII sites of vector pSPαIR-ZEO-αIR (28) and transfected by electroporation into the *L. infantum* wild type (*Li*DDX3^(+/+)^ or *Li*WT) and the *Li*DDX3^(−/−)^ (*Li*DDX3 KO) strains. The gene encoding the ATP-binding cassette protein subfamily E-member 1 (Rli1/ABCE1) (LINF_210012700) was PCR-amplified from *L. infantum* genomic DNA using specific primers to include an HA epitope-tag at the C-terminus (Supplementary Table S1), then cloned into the XbaI and HindIII sites of the puromycin acetyltransferase (PURO)-expressing vector pSPαIR-PURO-αIR (29) and transfected into the *Leishmania* WT and DDX3 KO strains. Stable transfectants were selected and cultivated with 0.6 mg/ml zeocin (Invitrogen) or 100 μg/ml puromycin (Invitrogen). To replace one of the two *L. infantum DDX3* genomic copies by DDX3-HA, the *DDX3* gene was PCR-amplified to include an HA epitope tag at the C-terminus and then cloned into XbaI and HindIII sites of vector pSPαIR-ZEO-αIR. The 5′- and 3′-flanking regions (∼300 bp each) necessary for homologous recombination were PCR-amplified from *L. infantum* genomic DNA and cloned into the HpaI and PvuII sites, respectively. The NotI sites at the extremities of the 5′ and 3′-flanking homologous regions (see Supplementary Table S1) were used to generate the linear cassette for transfections. The LINF_280035000 gene encoding the cytoplasmic (cyt) Hsp70 chaperone was PCR-amplified from *L. infantum* genomic DNA using specific primers (Supplementary Table S1) and cloned into XbaI and HindIII sites of vector pSPαIR-PURO-αIR and then transfected into the *Li*DDX3^(−/−)^ mutant. The open reading frame encoding the highly conserved ubiquitin (76 aa) was amplified from the *L. infantum* gene encoding ubiquitin fusion protein (LINF_310028300) with N-terminal 2xHA epitope sequences, cloned into XbaI and HindIII sites of pSPαIR-PURO-αIR and then transfected independently into *L. infantum* WT and DDX3 knockout strains.

### Protein lysate preparations, western blots and immunoprecipitations

Western blots were performed following standard procedures. The anti-mouse hemagglutinin (HA) tag monoclonal antibody (1:3000; ABM, Canada), the anti-rabbit-HA polyclonal antibody (SG77, Invitrogen) (1:500), anti-mouse FK2 monoclonal antibody recognizing mono- or poly-ubiquitin chains linked on target proteins via K29, K48 and K63 residues of ubiquitin (Enzo, Canada) (1:3000 and blocking in 1% BSA), the anti-Hsp70 (cytosolic) mouse monoclonal antibody universal (5A5-Alexis Biochemicals, Canada) (1:3000), the streptavidin-Horseradish peroxidase (HRP) antibody (Invitrogen, USA) (1:50 000), and the anti-*Leishmania* P0 (anti-rabbit, 1:3000) antibody (kindly provided by Dr Osvaldo de Melo Neto, Instituto Aggeu Magalhães, Fundação Oswaldo Cruz, Recife, Brazil) were used in these studies. Proteins isolated from 15% to 45% sucrose gradient fractions were precipitated with 20% trichloroacetic acid (TCA) (Sigma) and washed once with 100% acetone. The TCA-precipitated proteins from each fraction were lysed in 2xLaemmli buffer and resolved on SDS-PAGE. Blots were visualized by chemoluminescence with Pierce ECL2 western blotting kit (Thermo Scientific). Immunoprecipitation studies were carried out as described previously (23). Briefly, *L. infantum* promastigotes expressing HA-tagged proteins of interest were lysed, mixed with Pierce anti-HA magnetic beads (Thermo Scientific, Canada) and kept at –20°C prior to mass spectrometry analysis or alternatively resolved on SDS-PAGE, excised, trypsin-digested and analyzed by mass spectrometry.

### [^35^S] Methionine incorporation and pulse-chase assays

Exponentially grown *L. infantum* promastigotes (10^7^) were washed once with sterile 1× PBS and resuspended in 1 ml methionine-free RPMI-1640 media (Gibco, Thermo Fisher Scientific) reconstituted with 10% FCS, penicillin, streptomycin and glutamine and incubated at 25°C for 1 h. Two μCi [^35^S]-Met (Perkin Elmer) was added to the sample and cells were incubated for an additional 30 min. Cells were pelleted, washed twice with ice-cold phosphate-buffer saline (PBS) and lysed in SDS-PAGE 1× sample buffer without Coomassie blue (CBB). The [^35^S]-Met incorporated peptides were precipitated by TCA and [^35^S]-Met incorporation was measured in scintillation counter (Beckman LS 6000TA). For pulse-chase analysis, 6 × 10^7^ cells were washed once in 1× PBS, then resuspended in methionine-free media and incubated for 1 h in 6 ml followed by 10 min [^35^S]-Met pulse (20 μci/6 × 10^7^ cells). Immediately after 10 min, cells were washed once in 1× PBS and resuspended in 6 ml SDM-79 medium supplemented with 10% heat-inactivated FCS and samples were harvested (10^7^ cells) over 90 min at 15 min intervals. During chase, ^35^S-Met is competed by unlabelled methionine resulting in a decrease of the ^35^S signal. *De novo* protein synthesis was measured as ^35^S-Met incorporation for each time point, and values were normalized with the ^35^S signal of the initial 15 min time point (considered as 100%).

### Polysome analysis by density gradient fractionation

*L. infantum* promastigotes (2 × 10^9^) grown in SDM-79 medium (10% FCS) were harvested at OD_600_ around 0.250–0.280 and treated with 150 μg/ml cycloheximide for 15 min (10 min incubation followed by 5 min centrifugation). Cells were washed with 1xPBS containing 150 μg/ml CHX and lysed in 600 μl polysome lysis buffer [(10 mM Tris–HCl pH 7.4, 150 mM NaCl, 10 mM MgCl_2_, 1 mM DTT, 0.5% NP40, 100 μg/ml CHX, 40 U/ml RNAseOUT (Invitrogen), 1 mM PMSF, protease inhibitor tablet (Roche)] after passing through 1 ml syringe (6–8 times). Cytoplasmic lysates (RNA equivalent of 600 μg) were layered on top of 15–45% sucrose gradient (10 ml) in gradient buffer (50 mM Tris–HCl pH 7.4, 50 mM KCl, 10 mM MgCl_2_, 1 mM DTT, 4 U/ml RNAseOUT), as described previously (30). The total RNA concentration of the loaded extracts on each gradient was quantified using a Nanodrop (1μl of total *Leishmania* cell lysate was used for quantification). Ribosomal subunits (40S and 60S), monosomes (80S) and polysomes were sedimented by centrifugation in a Beckman SW40 Ti rotor at 35 000 rpm for 2.15 h at 4°C. Fractions were collected using an automated ISCO density fractionation system under continuous monitoring of the absorbance at 254 nm.

### Ribosome transit time measurements

The ribosomal half-transit time was measured as described previously (31) with slight modifications. Briefly, 10^8^ exponentially grown parasites for each time point (2–12 min) were washed with sterile 1xPBS and re-suspended in 1 ml methionine-free RPMI-1640 media at 25°C for 1 h. Approximately 3 μCi equivalent of [^35^S]-Met for each time point was added and parasites were harvested after 2 min intervals in 100 μg/ml CHX. Cells were washed twice in ice cold 1xPBS, lysed in 1 ml of polysome lysis buffer (10 mM Tris–HCl pH 7.4, 150 mM NaCl, 10 mM MgCl_2_, 1 mM DTT, 0.5% NP40, 100 μg/ml CHX, 40 U/ml RNaseOUT, 1 mM PMSF, protease inhibitor tablet) and centrifuged at 3000 rpm for 5 min to remove the organellar fractions and mitochondrial ribosomes. The lysate was cleared by centrifugation at 13 000 rpm and 4°C for 15 min to obtain the post-mitochondrial supernatant (PMS) fraction representing the total synthesized polypeptides (nascent still bound to the ribosome and completed polypeptides released from the ribosome). Part of the PMS (800 μl) was centrifuged at 90 000 g (30 000 rpm) in a Beckman ultracentrifuge at 4°C for 45 min to pellet ribosomes and collect the post-ribosomal supernatant (PRS) (ribosome-free) harboring only the released polypeptides. Proteins from PMS and PRS fractions were precipitated with ice-cold 20% TCA and [^35^S]-Met incorporation was measured by scintillation counting (Beckman). Plotting the values of [^35^S]-Met incorporation into PMS (total CPM) and PRS (released CPM) as a function of time (2–12 min) results in two straight lines. The PMS signal (total protein) is always higher than that of PRS (released polypeptides). The ribosomal half-transit time was obtained as the displacement in time (time delay) between the intercepts of the PMS (total CPM) and PRS (released CPM) lines on the time axis, which were determined by linear regression analysis as described previously (31). This parameter describes the average processivity of the ribosome.

### *In vitro* Bio-Puro conjugation assay and streptavidin pull-down of ubiquitinated polypeptides

To isolate ribosomes, 2 × 10^9^*Li*WT or *Li*DDX3^(−/−)^ promastigotes episomally expressing or not the HA-Ub plasmid were lysed in polysome lysis buffer and the lysate was cleared by centrifugation at 13 000 rpm for 10 min. The cleared lysate was layered onto 5 ml of 35% sucrose cushion prepared in sucrose cushion buffer (32) (10 mM Tris–HCl pH 7.4, 85 mM KCl, 5 mM MgCl_2_) and centrifuged at 50 000 rpm for 2 h in a 70.1 Ti Beckman rotor. The supernatant was discarded and washed once gently in RNase-free water without displacing the pellet, and the pellet was resuspended in 100 μl of Bio-Puro buffer (10 mM Tris–HCl pH 7.4, 400 mM KCl and 3 mM MgCl_2_)_._ For the bio-puromycylation reaction, 3 μM biotinylated puromycin (Jena Bioscience) was mixed with 2.5 A_260_ (∼100 μg of RNA) units of ribosomes in a 100 μl reaction and incubated at 25ºC for 90 min. After the bio-puromycylation reaction, 5% of each sample was analyzed to determine the total HA-ubiquitin conjugated proteins in the ribosome. The remaining sample was made up to 1 ml using Bio-Puro buffer and loaded onto 10 μl HA-conjugated Pierce anti-HA magnetic Dynabeads and rotated at 4°C overnight with gentle rotation. The sample was washed gently six times with 1× PBS–Tween (15 min each) and resuspended in 2× Laemmli buffer by boiling at 95°C for 5 min and resolved in 10% SDS PAGE gel, immunoblotted to Immobilon-P polyvinylidene difluoride (PVDF) membrane (Millipore) and the membrane was blocked in 2% BSA in 1× TBS-T buffer at room temperature for 30 min. The HA-Ub conjugated nascent polypeptides were detected by incubating with streptavidin-HRP antibody in the same buffer for 1 h followed by three 5 min washes. To detect the HA-Ubiquitinated proteins, the same membrane was hybridized with anti-rabbit HA antibody in 2% BSA in TBS-T. For the detection of endogenous ubiquitinated nascent chains, the 100 μl reaction which consists of bio-puromycylated ribosomes was incubated overnight at room temperature with streptavidin agarose beads (EMD Millipore Corp., USA) and 1 ml high stringency urea wash buffer (8 M urea /2% SDS/100 mM Tris–HCl and 150 mM NaCl) with gentle rotation. The sample was centrifuged at 2000 rpm for 2 min and the four 20 min washes were performed as described in (33) followed by a high salt buffer wash (100 mM Tris–HCl pH 7.4 and 1M NaCl) (for ribosome dissociation) and washed twice with high-purity water. The loose streptavidin agarose-nascent chain pellet was resuspended in 60 μl 2× Laemmli buffer and boiled at 95°C for 5 min. Ubiquitinated nascent chains were detected by western blotting using anti-ubiquitin FK2 antibody and the same membrane was blocked in 2% BSA TBS-T overnight and hybridized with streptavidin HRP antibody.

### Isolation of cytosolic protein aggregates

Protein aggregates were analyzed essentially as described previously (34) with slight modifications. Equal numbers (3 × 10^8^) of exponentially grown *L. infantum* (OD_600_ 0.300) were harvested and washed once with 1× PBS. The pellets were resuspended in 500 μl lysis buffer (50 mM potassium phosphate buffer pH 7.0, 1 mM EDTA, 5% glycerol, 1 mM PMSF) and lysed by freezing (30s) and thawing at 37°C (4–5 min) (5 times) with liquid nitrogen. Intact cells were removed by centrifugation at 3000 rpm at 4°C for 10 min and equal amount of protein was used in the subsequent steps. The insoluble membranes and aggregates were isolated by subsequent centrifugation at 13 000 rpm for 20 min. The pellet containing the membranes and aggregated proteins was resuspended in 320 μl of lysis buffer by brief sonication (level 2, 30 s). Membrane proteins and lipids were removed by adding 80 μl of 10% (v/v) NP40 and pelleted by centrifugation at maximum speed (13 000 rpm). This step was repeated twice and the NP40 insoluble protein aggregates were visualized by gel electrophoresis followed by silver staining.

### Label-free quantitative proteomic analysis

Three biological replicates of total protein lysates and also of ribosomes collected by 35% sucrose cushion centrifugation from *L. infantum* wild type and *Li*DDX3 knockout promastigotes were used for this analysis. Bands of interest were extracted from gels and placed in 96-well plates and then washed with water. Proteins were reduced with 10 mM DTT and alkylated with 55 mM iodoacetamide. Trypsin digestion was performed using 126 nM of modified porcine trypsin (Sequencing grade, Promega, Madison, WI) at 37°C for 18 h. Peptides were extracted using 1% formic acid, 2% acetonitrile followed by 1% formic acid, 50% acetonitrile. The recovered extracts were pooled, vacuum centrifuge dried and then resuspended into 10 μl of 0.1% formic acid, and 5 μl was analyzed by mass spectrometry. Samples were analyzed by nanoLC/MSMS using an Orbitrap Fusion mass spectrometer (Thermo Fisher Scientific, San Jose, CA, USA) connected to Dionex UltiMate 3000 nanoRSLC chromatography system (Thermo Fisher Scientific). Peptides were trapped at 20 μl/min in loading solvent (2% acetonitrile, 0.05% TFA) on a 5 mm × 300 μm C18 pepmap cartridge pre-column (Thermo Fisher Scientific) during 5 minutes. Then, the pre-column was switched online with a Pepmap Acclaim (Thermo Fisher) 50 cm × 75 μm separation column and the peptides were eluted with a linear gradient from 5–40% solvent B (A: 0.1% formic acid, B: 80% acetonitrile, 0.1% formic acid) for 90 min at 300 nl/min for a total length run of 120 min. Mass spectra were acquired using Thermo XCalibur software version 4.1.50. Full scan mass spectra (350–1800 *m*/*z*) were acquired in the Orbitrap using an AGC target of 4e5, a maximum injection time of 50 ms and a resolution of 120 000. Internal calibration using lock mass on the *m/z* 445.12003 siloxane ion was used. Each MS scan was followed by acquisition of fragmentation MS/MS spectra of the most intense ions for a total cycle time of 3 s (top speed mode). The selected ions were isolated using the quadrupole analyzer in a window of 1.6 *m/z* and fragmented by Higher energy Collision-induced Dissociation (HCD) with 35% of collision energy. The resulting fragments were detected by the linear ion trap in rapid scan rate with an AGC target of 1e4 and a maximum injection time of 50 ms. Dynamic exclusion of previously fragmented peptides was set for a period of 30 s and a tolerance of 10 ppm.

### Data analysis

Spectra were searched against the *L. infantum* database (8591 entries) (TriTrypDB version 41 released 13 December 2018; https://tritrypdb.org/) using the Andromeda module of MaxQuant software v. 1.6.0.16. Trypsin/P enzyme parameter was selected with two possible missed cleavages. Carbamidomethylation of cysteines was set as fixed modification while methionine oxidation was set as variable modifications. Mass search tolerance was 5 ppm and 0.5 Da for MS and MS/MS, respectively. For protein validation, a maximum False Discovery Rate of 1% at peptide and protein level was used based on a target/decoy search. MaxQuant was also used for Label-free quantification. The ‘match between runs’ option was used with 20 min value as alignment time window and 0.7 min as match time window. Only unique and razor peptides were used for quantification. RStudio 1.2.5019 was used for data processing. A normalization step was performed using the median of the median intensities of each condition. When some peptide values were missing, there were replaced by a noise value corresponding to 1% of the normalized value for each condition. A peptide was considered as *quantifiable* only if at least three intensity values in one of the two conditions were present. Only proteins identified with at least 2 peptides were considered (*quantified proteins*). For each protein in each comparison, the following values were calculated: the mean intensity in each group (mean g1 et mean g2), the intensity ratio between each group (ratio g1/g2), the log_2_ of g1/g2 ratio (log_2_ratio), the *z*-score *z* = *x* – mean/standard deviation (zscore), and the Limma *P-value* and *q-value* (Benjamini Hochberg adjusted *P-value*). To be significantly differentially expressed, a protein has to have a *q*-value less than 0.01 and a *z*-score ≤–1.96 and ≥1.96 for downregulated and upregulated proteins, respectively.

## RESULTS

### The *Leishmania* DDX3 ortholog co-sediments with the ribosome

**Figure 1.**
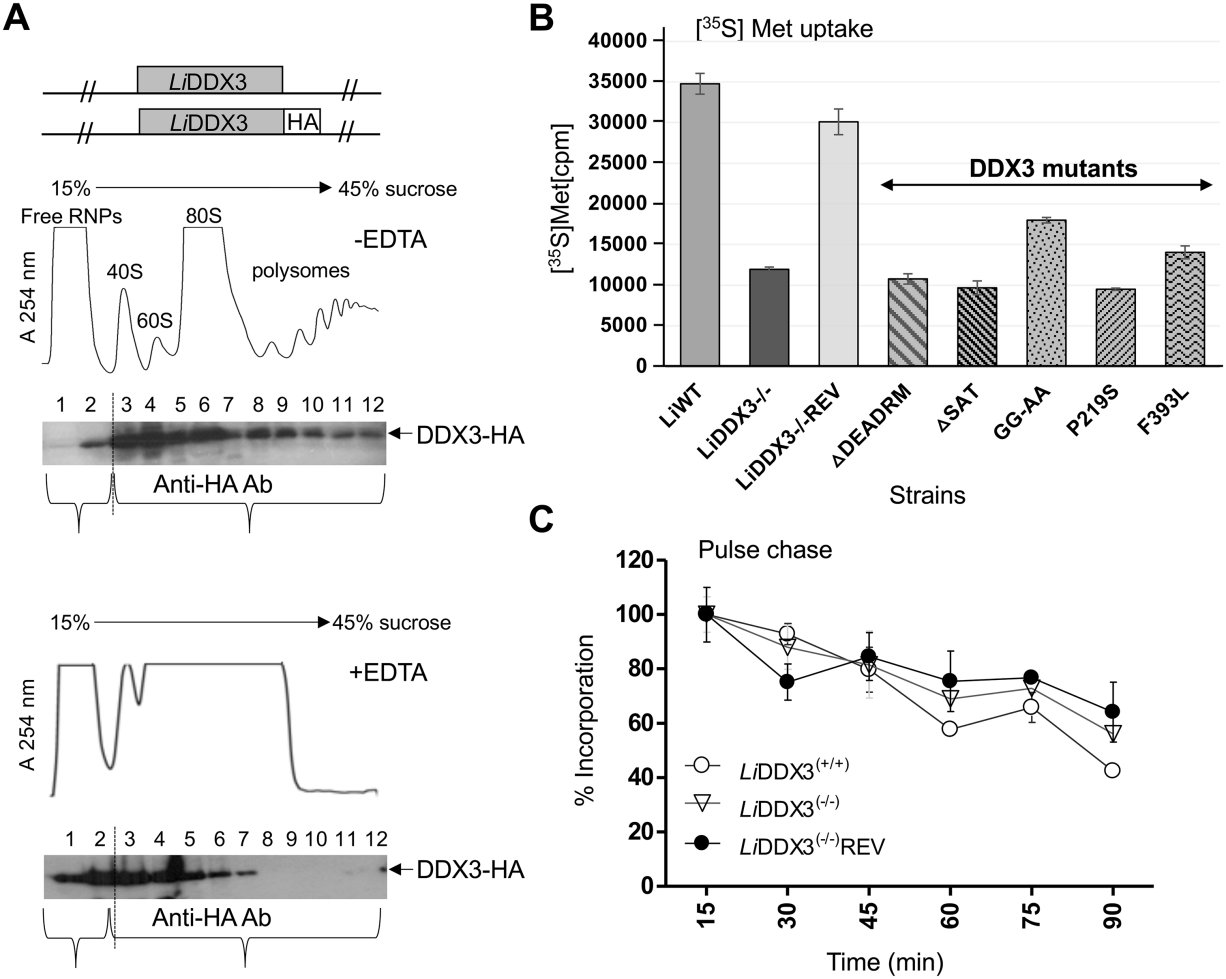
The *Leishmania* DDX3 ortholog is associated with translating ribosomes and its genetic depletion slows down *de novo* protein synthesis. (**A**) Schematic diagram of HA-epitope tagged *Li*DDX3 (*Li*DDX3-HA) used to replace one of the two *L. infantum DDX3* genomic alleles (upper panel). Ribosomal distribution of DDX3 as determined by polysome analysis of *L. infantum* WT cells (lower panel). Cytoplasmic lysates were layered on top of a linear 15–45% sucrose gradient and sedimented following ultracentrifugation to allow the separation of free ribonucleoprotein complexes (RNP) particles from 40S and 60S ribosomal subunits, 80S monosomes, and polysomal fractions according to their respective densities with the continuous absorbance measurement at 254 nm without (middle panel) and with EDTA (50 mM) (lower panel). Trichloroacetic acid (TCA) precipitated proteins from sucrose gradient fractions were separated on 10% SDS-PAGE gels and subjected to western blot analysis with anti-HA antibody. The quantification was done with ImageJ software. (**B**) [^35^S] Met uptake in *L. infantum* wild type (*Li*WT), DDX3 knockout (*Li*DDX3^(−/−)^), add-back mutant (*Li*DDX3^(−/−)^REV) and *Li*DDX3^(−/−)^ recombinant strains ectopically expressing different DDX3 mutant proteins with deletions or amino acid substitutions within conserved signature motifs involved in ATP binding /hydrolysis or RNA binding (see also Supplementary Figure S1). [^35^S]-Met incorporated peptides corresponding to *de novo* synthesized proteins were measured in a scintillation counter after TCA precipitation and expressed as incorporation percentage. (**C**) Pulse-chase analysis using *Li*WT, *Li*DDX3^(−/−)^ and *Li*DDX3^(−/−)^REV strains. Parasites grown in methionine-free medium were first subjected to [^35^S]-Met pulse. During the 90 min chase with 15 min intervals, ^35^S-Met is competed by unlabeled methionine resulting in a decrease of the ^35^S signal. *De novo* protein synthesis was measured as ^35^S-Met incorporation for each time point, and values were normalized with the ^35^S signal of the initial 15 min time point (considered as 100%). The results shown in B and C are represented as the mean of three independent experiments. Error bars indicate the standard deviation of the mean.

Our previous studies have shown that the *Leishmania* ortholog of DEAD-box RNA helicase DDX3 (*Li*DDX3) (LINF_320009100; http://tritrypdb.org/tritrypdb/) protects ribosomal RNA from degradation under conditions of stress or drug-induced cell death (27), hence suggesting a role of this helicase in ribosome homeostasis. To confirm ribosomal association of *Li*DDX3, we carried out polysome analysis using 15–45% sucrose gradient fractionation of *Leishmania infantum* recombinant cells expressing an HA-tagged *Li*DDX3 version *(Li*DDX3-HA) integrated into the *LiDDX3* endogenous locus (Figure 1A). Immunoblotting of trichloroacetic acid (TCA)-precipitated sucrose gradient fractions (40S and 60S ribosomal subunits, 80S monosome and polysomes) with anti-HA antibody revealed that the vast majority (∼90%) of the *Li*DDX3-HA signal was associated with ribosomal fractions (Figure 1A, upper panel). Even after EDTA treatment allowing the dissociation of polysomes, the majority of *Li*DDX3-HA (75%) was still associated with ribosomes (Figure 1A, lower panel). Moreover, DDX3-HA immunoprecipitation (IP) from DDX3-HA expressing cells coupled to LC–MS/MS analysis showed that *Li*DDX3 co-immunoprecipitates the vast majority of ribosomal proteins and components of the translation machinery (Supplementary Table S2). Accordingly, immunofluorescence studies indicated cytoplasmic localization for *Li*DDX3 (Supplementary Figure S2).

### Genetic depletion of DDX3 decreases *de novo* protein synthesis and leads to changes in the expression of distinct ribosomal proteins and ribosome biogenesis factors

To investigate the role of *Li*DDX3 in translation regulation, we first carried out metabolic labeling experiments with [^35^S] Methionine ([^35^S]-Met) to follow the biosynthesis of proteins in *L. infantum* wild type (*Li*WT), *Li*DDX3^(−/−)^ knockout mutant (DDX3 KO) and add-back mutant ectopically expressing DDX3 tagged with an HA epitope at the C-terminus (*Li*DDX3^(−/−)^REV). For [^35^S]Met labeling as for any other experiment in this study we only used the promastigote life stage of the parasite as DDX3 knockout cells were unable to survive as axenic or intracellular amastigotes (23). Interestingly, *L. infantum* DDX3 KO parasites showed ∼3.0-fold decrease in [^35^S]-Met-labeled synthesized polypeptides as compared to the WT (Figure 1B and Supplementary Figure S3, upper panel). Reduced protein synthesis was due to the inactivation of DDX3 since translation rates were restored close to the WT levels in the add-back mutant (Figure 1B). Expression of the DDX3 paralog, Dbp1 (LINF_350036300), remained unchanged between WT and DDX3 KO cells as determined by label-free quantitative (LFQ) analysis of the total proteome of these strains (Supplementary Table S3). Although we cannot exclude overlapping functions between DDX3 and Dbp1, our data suggest that Dbp1 did not compensate (at least not fully) for the translation slowdown in cells lacking DDX3. LFQ analysis revealed only one ATP-dependent DEAD/H RNA helicase (LINF_360053300; 625 aa) which was upregulated in DDX3 KO cells (Table 1, Supplementary Table S3). LINF_360053300 did not co-immunoprecipitate with DDX3 (Supplementary Table S2) and its function in translation, if any, remains to be explored.

**Table 1.** Selected proteins differentially expressed between *L. infantum* (*Li*) wild type (WT) and DDX3 knockout (KO) strains identified by label-free quantitative (LFQ) analysis of the total proteome

*Li*DDX3 KO (g1) versus *Li*WT (g2)								
	Predicted function	Unique peptides	Ratio g1/g2*	Log2 ratio*	*z*-score*	*P*val Limma*	*Q*val Limma*	Sign zscore *q*val Limma
**Ribosomal proteins / Ribosome biogenesis factors**								
LINF_280031800	40S ribosomal protein S29 - putative	4	0.264	–1.921	–2.758	0.000144	0.002924	Down
LINF_360046400	40S ribosomal protein S27 - putative	7	0.298	–1.744	–2.509	7.78E–05	0.001980	Down
LINF_260021500	40S ribosomal protein S33 (S28e)- putative	8	0.162	–2.621	–3.750	3.84E–06	0.000421	Down
LINF_110014700	60S ribosomal protein L24 - putative	5	3.329	1.735	2.415	4.41E–06	0.000432	Up
LINF_100015300	Ribosome biogenesis protein Nop16	4	2.717	1.442	2.001	0.000157	0.003134	Up
LINF_290026100	Pre-RNA processing PIH1/Nop17- putative	2	6.823	2.770	3.881	1.46E–05	0.000770	Up
**Protein deubiquitination**								
LINF_160012700	Ubiquitin hydrolase - putative	9	5.986	2.581	3.613	0.004322	0.022190	Up
**DEAD-box RNA helicases**								
LINF_030011900	DEAD/DEAH box helicase/Type III restriction enzyme-res subunit - putative	39	0.389	–1.359	–1.964	8.98E–06	0.000594	Down
LINF_360053300	ATP-dependent DEAD/H RNA helicase	11	65.705	6.037	8.505	1.72E–08	1.63E–05	Up

For each protein, the following values were calculated: i) the intensity ratio between each group (ratio mean g1 et mean g2); ii) the log_2_ of g1 to g2 ratio (log_2_ ratio); iii) the *z*-score, *z* = *x* – mean/standard deviation (zscore); iv) Limma *P*-value and *q*-value (Benjamini Hochberg adjusted *P-value*), *q*-value < 0.01 and z-score (≤–1.96 and ≥1.96 for downregulated and upregulated proteins, respectively) (‘Sign_zscore_qval_Limma’). Only proteins identified with at least two peptides were considered. A more detailed list of selected up- or down-regulated proteins in the DDX3 knockout strain is shown in Supplementary Table S3 and the full list of identified proteins is presented in Supplementary Table S4.

To assess the contribution of distinct DDX3 ‘helicase core’ motifs (see Supplementary Figure S1) in translation regulation, we generated different DDX3 mutant protein versions and evaluated their capacity to rescue the *L. infantum* DDX3 KO strain. Deletion of motif II (LDEADRM) involved in ATP binding and hydrolysis (35) or of motif III (SAT) important for RNA-dependent hydrolysis of ATP (but not for ATP affinity) and single-stranded RNA binding (36) failed to rescue DDX3 KO cells (Figure 1B). A single P219S amino acid substitution within the PTREL motif (Ia) involved in RNA binding (35) or a F393L mutation within the FVE motif (IV) required for ATP hydrolysis and ATP-dependent binding of RNA substrates (37) failed to fully restore *de novo* protein synthesis (Figure 1B). A double GG to AA substitution within GG motif (Ib) involved in RNA binding only partially rescued DDX3 KO cells (Figure 1B). Collectively, these data indicate that both ATP- and RNA-binding activities of DDX3 are equally important for *Li*DDX3′s function in translation regulation.

To determine whether translation slowdown in the absence of DDX3 was due to decreased *de novo* protein synthesis or to enhanced protein degradation, we carried out pulse chase analysis. The results did not show any significant difference in the turnover of [^35^S]Met-labeled proteins between the DDX3 KO and control strains (Figure 1C), hence supporting reduced *de novo* protein synthesis in the absence of DDX3. While inactivation of *Li*DDX3 decreases *de novo* protein synthesis by ∼3-fold (Figure 1B and Supplementary Figure S3, upper panel), steady-state protein levels do not seem to be significantly reduced in DDX3 KO cells in comparison to the controls (see CBB staining in Supplementary Figure S3). Quantitative proteomic analysis corroborates these data as only 1.4% of the total proteome was downregulated in the DDX3 KO strain (40 proteins out of the 2850 identified were downregulated; see Supplementary Table S4). These results suggest that upon DDX3 loss ribosomes synthesize proteins at a slower pace than WT cells, which also explains the slower growth of DDX3 KO cells.

With regards to decreased protein synthesis upon DDX3 depletion, LFQ analysis of the total proteome revealed that the 40S subunit proteins S27, S29 and S33 (S28e) were downregulated in the DDX3 KO mutant (Table 1 and Supplementary Table S3). S27 and S29 may be direct targets for DDX3, as RNA-seq analysis showed that their corresponding transcripts were also downregulated in DDX3 KO cells (Zghidi-Abouzid *et al.*, unpublished). Downregulation of these 40S ribosomal subunit proteins may also contribute to translation slowdown in DDX3 KO cells. By contrast, the 60S L24 protein was upregulated in DDX3 KO cells (Table 1 and Supplementary Table S3). L24 resides on the surface of the 60S subunit and it was shown previously to regulate joining of the 60S to the 40S subunit (38) and to stimulate *in vitro* translation initiation and elongation in *S. cerevisiae* (39). Thus, upregulation of L24 may compensate for the decreased protein synthesis in DDX3 KO cells. Moreover, ribosome biogenesis factors Nop16 and Nop17 participating in pre-rRNA processing (40) were upregulated in DDX3 KO cells (Table 1 and Supplementary Table S3) possibly to counterbalance for the higher levels of rRNA degradation described previously in these cells (27).

### Translating ribosomes do not elongate optimally in the absence of DDX3

**Figure 2.**
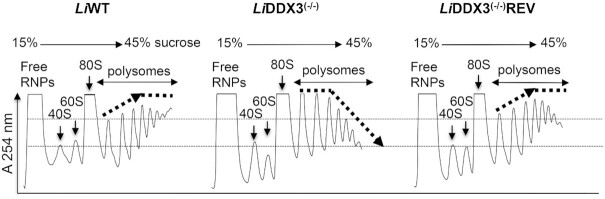
Translating ribosomes do not elongate optimally in the absence of DDX3. Cytoplasmic lysates from *L. infantum* WT (*Li*WT), *Li*DDX3^(−/−)^ and *Li*DDX3^(−/−)^REV strains (equal amount of RNA 600 μg was used for all strains) treated briefly with cycloheximide (CHX) prior to fractionation were loaded on linear 15–45% sucrose gradients and fractionated by ultracentrifugation by continuously recording absorbance (A) at 254 nm to separate 40S and 60S ribosomal subunits from 80S and polysome fractions. The density of 2- to 4-ribosome containing fractions is significantly higher in DDX3 knockout cells in comparison to the WT and rescue strains. This profile indicates that leading ribosomes advance more slowly in cells lacking DDX3 so that following ribosomes cannot progress, which infers that elongating ribosomes encounter problems and stall. By contrast, the 5- to 8-ribosome containing fractions (heavy polysomes) sharply decrease in the DDX3 knockout mutant as opposed to the controls and progressively drop-off from the mRNA template consistent with impaired translation. The data shown here are representative of at least four independent experiments with similar results (see also Supplementary Figure S4).

To further our investigation on the role *Li*DDX3 plays in translation regulation in *Leishmania*, we first carried out polysome analysis using sucrose density gradient ultracentrifugation. This technique allows to separate and visualize actively translating ribosomes from non-translating fractions and to test for perturbations in the various steps of translation. Equal numbers of exponentially grown parasites from WT, DDX3 KO and rescue strains were lysed similarly and lysates (equal amount of RNA 600 μg) were layered on top of a 15–45% sucrose gradient and subjected to ultracentrifugation to separate mRNP complexes by velocity sedimentation, as detailed in Materials and Methods. Although there were no significant changes in the peaks of free 40S and 60S ribosomal subunits and monosomes (80S) between the three strains, the polysome distribution was different in DDX3 KO cells (Figure 2). In fact, the peaks of light polysome fractions (2- to 4-bound ribosomes on mRNA) were significantly higher in DDX3 KO cells in comparison to the control strains, suggesting an increased number of ribosomes on the mRNA in these fractions. This could arise when a trailing ribosome encounters a slower leading ribosome and is an indication of ribosome stalling. By contrast, heavy polysomes (5- to 8-bound ribosomes on mRNA) sharply decreased in DDX3 KO cells and progressively dropped off from the mRNA template (Figure 2 and Supplementary Figure S4, middle panel). A similar trend was observed in the absence of cycloheximide (CHX), a drug that freezes elongating ribosomes, albeit as expected polysome peaks were lower both in WT and DDX3 KO strains (Supplementary Figure S4, upper panel). Treatment with harringtonine (HAR), a drug that immobilizes ribosomes selectively at initiation codons without altering those already engaged in elongation (41), also confirmed that there were globally less ribosomes engaged in the elongation process in cells lacking DDX3 (Supplementary Figure S4, lower panel). As expected, a higher monosome peak and gradual decrease in polysome peaks were also seen in WT HAR-treated cells (Supplementary Figure S4, lower panel). However, the effect on heavy polysome reduction was much more pronounced in cells lacking DDX3 (Supplementary Figure S4, lower panel) in line with the +/- CHX results (Figure 2). Polysome analysis in the presence of HAR suggested that ribosome stalling was largely reversible in DDX3 KO cells. Indeed, only the 2-ribosome containing fraction showed some retention (Supplementary Figure S4, lower panel). Collectively, polysome analysis revealed that upon DDX3 loss ribosomes accumulate and stall early in the elongation process. Although stalling seems to be largely reversible, translating ribosomes are less engaged in elongation and drop-off gradually from the mRNA. Stalling of early ribosomes can affect translation elongation by other ribosomes leading to reduced translation elongation. In line with the polysome data indicating perturbations in translation elongation in the absence of DDX3, the DDX3 KO mutant was more sensitive than WT and add-back strains to known inhibitors targeting translation elongation, such as puromycin dihydrochloride (PURO) causing a premature chain termination and ribosome release (Supplementary Figure S5A) and CHX (Supplementary Figure S5B).

### Ribosome speed along the mRNA slows down in cells lacking DDX3

**Figure 3.**
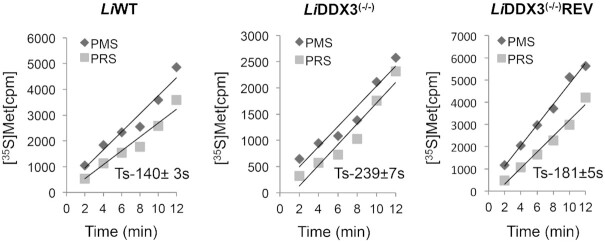
Ribosome movement along the mRNA slows down upon DDX3 depletion. Ribosome transit time measurements in *Li*WT, *Li*DDX3^(−/−)^ and *Li*DDX3^(−/−)^REV (rescue) strains were determined by calculating the kinetics of [^35^S]-Met incorporation into total protein (nascent and released polypeptides) in post-mitochondrial supernatant (PMS) and into completed polypeptides released from the ribosome in post-ribosomal supernatant (PRS), as detailed in Materials and Methods. The ribosomal half-transit time was obtained as the displacement in time between the intercepts of the PMS (total CPM) and PRS (released CPM) lines on the time axis, which were determined by linear regression analysis. The ribosomal transit time (Ts) in seconds is expressed as mean ± SD of two independent experiments. Two additional experiments showed a similar trend (see also Supplementary Figure S6).

To assess whether decreased *de novo* protein synthesis in DDX3 KO cells results from a slowdown in ribosome movement, we evaluated ribosome speed along the mRNA by measuring the ribosomal transit time which refers to the time it takes for a ribosome, after initiation, to run across an average-sized mRNA and release the fully synthesized polypeptide chain (42). Ribosome transit time measurements in *L. infantum* WT, DDX3 KO and rescue strains were determined by calculating the kinetics of [^35^S]-Met incorporation into total protein (nascent still bound to the ribosome and released) in post-mitochondrial supernatant (PMS) and into completed polypeptides released from the ribosome in post-ribosomal supernatant (PRS), as detailed in Materials and Methods. The average half-transit time was obtained from the displacement in time between the intercepts of the PMS (total CPM) and PRS (released CPM) lines on the time axis, which were determined by linear regression analysis as described previously (31) (see also Figure 3). If ribosomes move slower along the mRNA in the absence of DDX3, the DDX3 KO ribosomal transit time should be greater than that of the WT. Indeed, the estimated ribosomal half-transit time (Ts) was ∼1.7-fold longer in cells lacking DDX3 (239 ± 7 s) as compared to the controls (WT: 140 ± 3 s and DDX3^(−/−)^REV: 181 ± 5 s) (Figure 3), supporting that DDX3 inactivation significantly reduces the rate of polypeptide synthesis by elongating ribosomes. As expected, a DDX3 mutant lacking motif II (*Li*DDX3ΔLDEADRM) that is essential for ATP-binding and hydrolysis (35) failed to restore the decrease in ribosome speed (∼280s) once transfected into the DDX3 KO background (Supplementary Figure S6). The slower movement of translating ribosomes in cells lacking DDX3 corroborates our polysome analysis data supporting ribosome stalling and reduced engagement of translating ribosomes in the elongation process (Figure 2 and Supplementary Figure S4, upper and middle panels).

### The ribosome recycling factor Rli1/ABCE1 and translational GTPases eRF3 and GTPBP1 are less recruited to ribosomes in the absence of DDX3

To identify putative changes in the total proteome or the recruitment of translation and other ribosome-associated factors to ribosomes of cells lacking DDX3 that could possibly explain slowdown of ribosome speed leading to stalling, we carried out extensive LFQ mass-spectrometry analysis. The total proteome from WT and DDX3 KO strains (Supplementary Tables S3 and S4) but also polypeptides still associated with ribosomes (Supplementary Tables S5 and S6) collected from the same set of strains were analyzed. Quantification was done based on the relative intensity of unique peptides (at least two peptides) from three biological triplicates for each strain upon trypsin digestion, as detailed in Materials and Methods. This analysis revealed that the majority of ribosomal proteins, ribosome biogenesis, and translation factors identified remained unchanged between WT and DDX3 KO cells. However, some interesting differences were observed, as detailed below.

**Figure 4.**
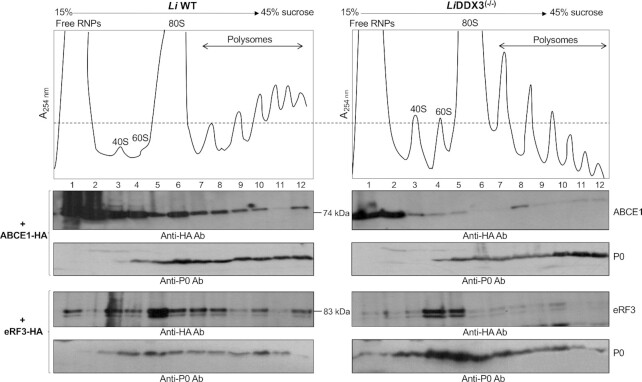
The recycling factor Rli1/ABCE1 and the GTP-bound release factor eRF3 are less recruited to the ribosome in the absence of DDX3. Ribosomal distribution of ABCE1 (upper panels) and eRF3 (lower panels) in *Li*WT and *Li*DDX3^(−/−)^ knockout cells. Cytoplasmic lysates from *L. infantum* WT and *Li*DDX3^(−/−)^ cells episomally expressing HA-epitope-tagged ABCE1 or eRF3 (equal amount of RNA 600 μg was used for both strains) were layered on top of a linear 15–45% sucrose gradient and sedimented following ultracentrifugation to allow the separation of free RNPs from 40S and 60S ribosomal subunits, 80S and polysomal fractions according to their respective densities. The different fractions were subjected to western blot analysis with anti-HA antibody. Western blots for *Li*WT and *Li*DDX3^(−/−)^ recombinant cells expressing ABCE1-HA or eRF3-HA were processed in parallel and exposure times were similar. Immunoblot with an antibody recognizing the *Leishmania* 60S ribosomal protein P0 was used as loading control. The quantification was done with ImageJ software. Polysome profiles and immunoblots shown here are representative of three independent experiments for ABCE1-HA and two independent experiments for eRF3-HA with similar results.

LFQ proteomic analysis of purified ribosomes by 35% sucrose cushion centrifugation revealed that the ATP-binding cassette subfamily E member 1 (ABCE1 in mammals)/Rli1 (ribonuclease L inhibitor in yeast) ortholog in *Leishmania* (LINF_210012700; 63% aa sequence identity with its human ortholog) was less recruited to the ribosome in DDX3 KO cells as compared to the WT (Table 2, Supplementary Table S5). The essential, conserved Rli1/ABCE1 ATPase uses the power generated from ATP-binding and hydrolysis to split terminating 80S ribosomes into the 40S and 60S subunits and recycle them for participation in new rounds of translation initiation (43). In addition, Rli1/ABCE1 can split and rescue vacant or inactive 80S ribosomes (44,45) or stalled ribosome complexes on truncated/aberrant mRNAs in concert with Dom34(yeast)/Pelota(mammals) and Hbs1(yeast)/HBS1L(mammals), the paralogs of eukaryotic release factors eRF1 and eRF3, respectively (44–47). To validate LFQ data on DDX3-dependent differential ribosomal association of ABCE1, we carried out polysome analysis of WT and DDX3 KO cells ectopically expressing ABCE1 tagged with an HA epitope at the C-terminus (Supplementary Figure S7A) followed by western blotting with anti-HA antibody. This experiment confirmed the lower recruitment of ABCE1 to DDX3 KO ribosomes in comparison to WT cells (Figure 4). Decreased ribosomal recruitment of ABCE1 was not due to differential expression of this protein between WT and DDX3 KO cells as similar protein levels were depicted by LFQ analysis of the total proteome (Supplementary Table S3).

**Table 2.** Selected proteins exhibiting differential recruitment to ribosomes between *L. infantum* (*Li*) wild type (WT) and DDX3 knockout (KO) strains identified by label-free quantitative (LFQ) analysis of ribosomes isolated by ultracentrifugation through 35% sucrose cushion.

			*Li*DDX3 KO (g1) versus *Li*WT (g2)					
Protein IDs (TriTrypDB)	Predicted function	Unique peptides	Ratio g1/g2*	Log2 ratio*	*z*-score*	*P*val Limma*	*Q*val Limma*	Sign zscore *q*val Limma
**Ribosome recycling / Ribosome rescue**								
LINF_210012700	ATP-binding cassette protein subfamily E member 1 (ABCE1/Rli1) - putative	3	0.099	–3.328	–1.980	0.000566	0.003701	Down
LINF_330037200	GTP-binding elongation factor Tu family protein-putative (GTPBP1)	3	0.101	–3.304	–1.967	0.001389	0.006873	Down
**Protein ubiquitination and proteasomal degradation**								
LINF_350029800	Ubiquitin-protein ligase (HECT type) -putative	11	61.61	5.945	2.865	5.16E–05	0.000749	Up
LINF_040011900	Ubiquitin-conjugating enzyme E2 - putative	3	29.62	4.888	2.313	6.83E–06	0.000211	Up
LINF_350022600	Proteasome complex subunit Rpn13 ubiquitin receptor - putative (ADRM1)	6	12.46	3.639	1.660	0.008848	0.024990	Up
LINF_240009000	Ubiquitin carboxyl-terminal hydrolase-putative (UCH37 deubiquitinating enzyme)	5	14.74	3.882	1.787	0.002149	0.009229	Up
LINF_050009100	Tetratricopeptide repeat/TPR repeat-putative Anaphase promoting complex-sub 3 (CDC27)	14	16.85	4.075	1.888	0.008909	0.025127	Up
LINF_300012300	CDC16-putative Anaphase-promoting complex-subunit 6	8	25.85	4.692	2.210	0.000673	0.004139	Up
LINF_040009000	Anaphase promoting complex - subunit 10-like	4	75.75	6.243	3.020	7.06E–06	0.000212	Up
**Chaperones**								
LINF_330016300	Dnaj chaperone-like protein	4	99.37	6.634	3.225	8.64E–08	3.52E–05	Up

For each protein, the following values were calculated: i) the intensity ratio between each group (Ratio mean g1 et mean g2); ii) the log2 of g1 to g2 ratio (log_2_ ratio); iii) the *z*-score, *z* = *x* – mean/standard deviation (zscore); iv) Limma *P*-value and *q*-value (Benjamini Hochberg adjusted *P-value*), *q*-value < 0.01 and *z*-score (≤–1.96 and ≥1.96 for downregulated and upregulated proteins, respectively) (‘Sign_zscore_qval_Limma’). Only proteins identified with at least two peptides were considered. A list of selected proteins related to ribosome function and translation is shown in Supplementary Table S5 and the full list of identified proteins is shown in Supplementary Table S6.

Furthermore, our LFQ proteomic data revealed no changes in the expression (Supplementary Table S3) or ribosomal recruitment (Supplementary Table S5) of release factor eRF1 and its paralog Dom34 between WT and DDX3 KO cells. The release factor eRF3 forming a ternary complex with eRF1 which enters the ribosome once the stop codon is reached to allow canonical translation termination and also ribosome recycling (48,49) was similarly expressed between WT and DDX3 KO cells (Supplementary Table S3) but not detected in the LFQ analysis of DDX3 KO ribosomes (Supplementary Table S6). We therefore investigated ribosomal association of eRF3 (LINF_110017700) in the absence of DDX3 using sucrose gradient fractionation and western blot analysis. Interestingly, as also seen for ABCE1, eRF3 was less recruited to ribosomes in the absence of DDX3 (Figure 4) (see also Supplementary Figure S7A for eRF3-HA expression). In addition, LINF_330037200 annotated as GTP-binding elongation factor Tu family protein was less recruited to DDX3 KO ribosomes (Table 2, Supplementary Table S5), albeit no differences were observed in the expression of this protein between WT and DDX3 KO strains (Supplementary Table S3). LINF_330037200 is the closest homolog of the human GTP-binding protein 1 (GTPBP1) (43% sequence identity), an unconventional translational GTPase closely related to eRF3 and Hbs1 involved in translation elongation and mRNA surveillance (50,51). Altogether, our results that Rli1/ABCE1, eRF3, and GTPBP1 are less recruited to ribosomes of cells lacking DDX3 suggest that arrested ribosomes may be not efficiently dissociated from the mRNA and recycled, which further explains slower ribosome translocation and translation elongation in these cells.

ABCE1-HA IP and mass-spectrometry analysis revealed possible interactions of ABCE1 with eRF1 (LINF_270024100) and eRF3 (LINF_110017700) but ABCE1 did not pull-down the eRF1 paralog Dom34/Pelota (LINF_260007000) (Supplementary Table S7). As opposed to other eukaryotes, the *Leishmania* genome does not encode the eRF3 paralog Hbs1. The *Leishmania* ABCE1 protein also co-immunoprecipitates most ribosomal components and translation factors, as well as several DEAD/DEAH-box RNA helicases including DDX3 (Supplementary Table S7). Inversely, DDX3-HA pulls-down ABCE1, eRF1 and eRF3 (Supplementary Table S2).

### Cells lacking DDX3 exhibit increased ubiquitination on the ribosomes

**Figure 5.**
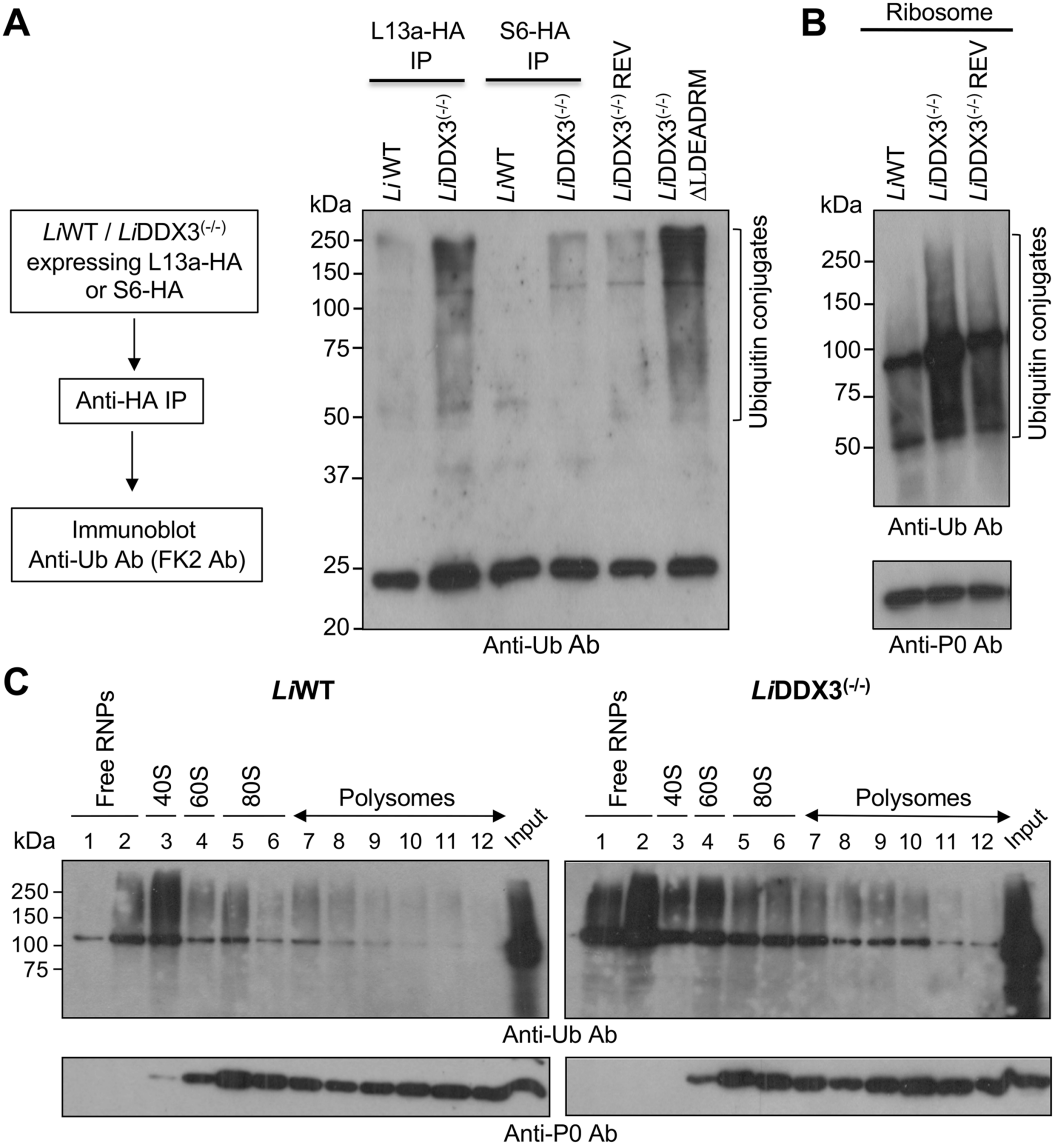
Cells lacking DDX3 display increased ubiquitination at the ribosome. (**A**) Ribosomal proteins of the large (L13a) and small (S6) subunits were used for immunoprecipitation (IP) studies to pull down ribosomal proteins and ribosome-associated factors (left panel). *Li*WT and *Li*DDX3^(−/−)^ strains ectopically expressing the C-terminally HA-epitope tagged ribosomal protein L13a or S6 were subjected to IP with anti-HA antibody followed by western blotting with anti-Ub (FK2) antibody recognizing mono- or poly-ubiquitin chains linked on target proteins via K29, K48 and K63 (right panel). Also shown here the DDX3 IPs from DDX3 KO cells complemented either with DDX3-HA (add-back mutant) or with the mutant protein DDX3ΔLDEADRM-HA (unable to rescue). The intense band at ∼25 kDa corresponds to the contaminant immunoglobulin light chain which can serve as loading control here. (**B**) Immunoblotting of intact ribosomes isolated from *Li*WT and *Li*DDX3 knockout cells by centrifugation through 35% sucrose cushion using anti-Ub antibody. Immunoblot with an antibody recognizing the *Leishmania* 60S ribosomal protein P0 was used as protein loading control. (**C**) Polysome analysis of *Li*WT and *Li*DDX3^(−/−)^ strains using sucrose gradient fractionation and western blotting with FK2 antibody to visualize the distribution of ubiquitinated proteins across the gradient. A similar amount of proteins from *Li*WT and *Li*DDX3^(−/−)^ strains was loaded for 60S, 80S and polysome-containing fractions as per the P0 antibody control. Blots shown in A-C are representative of two independent experiments with similar results.

Our data support ribosome stalling early in the elongation and slower speed of translating ribosomes upon DDX3 loss (Figures 2 and 3, Supplementary Figures S4 and S6). Ribosomes stall as a consequence of aberrant/truncated mRNAs and nascent polypeptides which have to be eliminated through ubiquitination and proteasomal degradation (5). To further our investigation on ribosome-associated ubiquitination in cells lacking DDX3, we carried out anti-Ub immunoblots on lysates enriched for ribosomal proteins, as well as on intact ribosomes. First, we did pull-downs with whole cell lysates from WT and DDX3 KO cells ectopically expressing HA-tagged ribosomal proteins 60S L13a (LINF_150007100) or 40S S6 (LINF_150022800) (Figure 5A left panel, Supplementary Figure S8A) to enrich for ribosomal proteins and associating factors. The homologs of these proteins in yeast (RpL16) (52) and mammals (RpS6) (53) have been used successfully for ribosomal protein pull-downs. LC-MS/MS analysis confirmed the presence of most 40S and 60S ribosomal proteins (69 out of 79) in L13a-HA and S6-HA pull-downs together with translation and other ribosome-associated factors (Supplementary Table S8). Western blot analysis with anti-ubiquitin (Ub) antibody FK2 recognizing mono- or poly-ubiquitin chains linked on target proteins via K29, K48 and K63 depicted higher ubiquitination in L13a-HA pull-downs from DDX3 KO lysates in comparison to the WT. The S6-HA pull-down showed also more ubiquitination than the WT, albeit at a lesser degree (Figure 5A right panel and Supplementary Figure S8B). Although ribosomal proteins are highly enriched in these pull-downs (Supplementary Table S8), our current data do not allow us to conclude if distinct ribosomal proteins, including L13a and S6, are ubiquitinated upon DDX3 depletion.

Next, we carried out anti-Ub (K29-, K48- and K63-linked chains) western blots of intact ribosomes isolated by ultracentrifugation through 35% sucrose cushion from *L. infantum* WT, DDX3 KO and add-back mutant. Our results indicated increased ubiquitination at the ribosome in cells lacking DDX3 (Figure 5B). It is likely that a significant part of these ubiquitinated products correspond to nascent polypeptides modified with K48-linked chains accumulating on stalled ribosomes in the absence of DDX3. K48-ubiquitin is the most abundant linkage type and the canonical signal for proteasomal degradation (54). The ubiquitination signal on the ribosomes can also include proteins with K63- or K29-linked chains, which may correspond to ribosomal proteins or ribosome-associated factors as reported previously in other eukaryotes under conditions of impaired translation (55). Moreover, ribosome fractionation by sucrose gradient sedimentation followed by anti-Ub western blotting similarly indicated higher labels of Ub-modified proteins in the DDX3 null mutant distributed across the gradient fractions (Figure 5C). Interestingly, more ubiquitination was detected on the 60S subunit as also shown in Figure 5A. Altogether, these data indicate that DDX3 loss results in higher levels of ribosome-associated ubiquitination. Increased ubiquitination was directly related to DDX3 loss as demonstrated by independent DDX3 IP studies from DDX3 KO cells complemented either with DDX3-HA (add-back mutant) or DDX3ΔLDEADRM-HA mutant (unable to rescue). A higher ubiquitination pattern was detected only when DDX3 could not be rescued (Figure 5A, Supplementary Figure S8B).

### Loss of DDX3 leads to increased co-translational ubiquitination of nascent polypeptides

**Figure 6.**
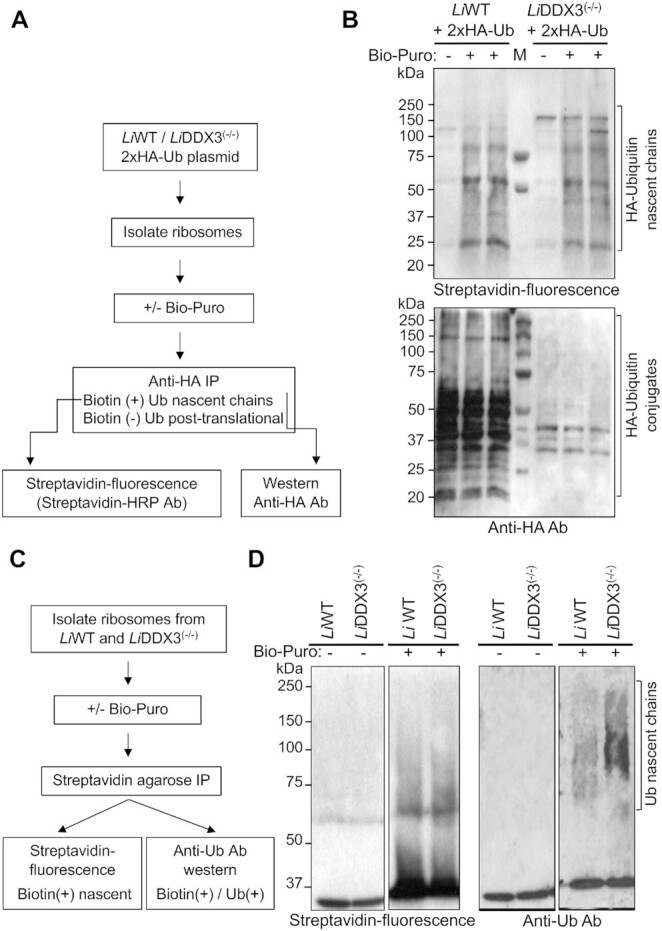
Cells lacking DDX3 exhibit increased co-translational ubiquitination of nascent polypeptide chains. (**A**) Schematic diagram of the *in vitro* biotin-puromycin (Bio-Puro) conjugation approach used to enrich for nascent polypeptide chains. Ribosomes from *Li*WT and *Li*DDX3^(−/−)^ strains stably transfected with a 2xHA-Ubiquitin (Ub) expressing plasmid were collected by ultracentrifugation through 35% sucrose cushion and incubated *in vitro* with Bio-Puro. (**B**) Biotin-labeled 2xHA-Ub conjugated nascent polypeptides were isolated by HA-magnetic beads pull-down and visualized with fluorescently tagged streptavidin (streptavidin-HRP antibody) (top panel). The same membrane was used for immunoblotting with anti-HA antibody to detect the HA-ubiquitin conjugated polypeptides (both biotin^+^ and biotin^–^) (bottom panel). The copy number of the 2xHA-Ub plasmid was ∼4-fold higher in *Li*WT as compared to the *Li*DDX3^(−/−)^ strain as determined by Southern blot hybridization (see Supplementary Figure S9A, B). Representative data from one out of three independent experiments with similar results are shown here (see also Supplementary Figure S9C). (**C**) A schematic diagram of an alternative approach to evaluate the presence of endogenous ubiquitin conjugates in untransfected *Leishmania* cells. (**D**) Similarly, ribosomes collected from *Li*WT and *Li*DDX3^(−/−)^ cells were incubated with Bio-Puro to label associated nascent chains, and total reaction products were isolated by streptavidin-agarose beads pull-down and immunoblotted with either streptavidin HRP antibody (biotin + nascent polypeptides; showing equal pull-downs of nascent peptides from both *Li*WT and DDX3 KO strains) or anti-ubiquitin (FK2) antibody (showing ubiquitinated nascent chains). Representative data from one out of two independent experiments with similar results are shown.

To address whether newly synthesized polypeptides were ubiquitinated in DDX3 KO cells, we first carried out *in vitro* Biotinylated-Puromycin (Bio-Puro) conjugation assay on ribosomes from WT and DDX3 KO cells expressing a 2xHA-tagged ubiquitin (2xHA-Ub) plasmid. Puromycin is a structural analog of aminoacyl-tRNA that blocks translation by forming a covalent bond with the carboxyl-terminus of nascent polypeptides (56). Bio-Puro moieties incorporate only into nascent chains and can be readily detected by streptavidin-HRP antibody after their enrichment by IP using anti-HA antibody (Figure 6A). HA-Ub moieties were efficiently conjugated to *Leishmania* proteins as revealed by western blotting (Supplementary Figure S9A). The higher levels of HA-Ub conjugates in WT cells can be explained by the presence of ∼4.0-fold more copies of the 2xHA-Ub expressing plasmid in comparison to DDX3 KO cells, as determined by Southern blot hybridization (Supplementary Figure S9B). In *Leishmania*, it is not yet well understood how plasmid copy number is controlled and it is common to have copy number variations between stable transfectants selected under the same conditions (57).

Ribosomes collected by ultracentrifugation through 35% sucrose cushion from 2xHA-Ub expressing WT and DDX3 KO strains were subjected to *in vitro* Bio-Puro reaction followed by IP with HA conjugated streptavidin magnetic beads to enrich for HA-Ub modified polypeptides [both co-translationally (biotin+) and post-translationally ubiquitinated biotin(−)] (Figure 6A). The samples were then resolved on SDS-PAGE and immunoblotted with streptavidin-HRP antibody to detect only the HA-Ub conjugated nascent polypeptides (modified both with Bio-Puro and HA-Ub). Nascent chains were successfully labeled with biotin as the streptavidin-HRP signal was dependent upon the addition of Bio-Puro (Figure 6B, top panel and Supplementary Figure S9C, right top panel). Immunoblotting with fluorescently tagged streptavidin showed a broader pattern of co-translationally ubiquitinated polypeptides in DDX3 KO cells (Figure 6B, top panel, Supplementary Figure S9C, right top panel). The extent of HA-Ub conjugated nascent chains is in fact much higher in the absence of DDX3 if we consider that there is 4-fold less HA-Ub plasmid copies in DDX3 KO cells than in WT (Supplementary Figure S9B). This was also confirmed by immunoblotting the same membrane with anti-HA antibody recognizing both biotin(+) and biotin(−) labelled polypeptides (Figure 6B, low panel and Supplementary Figure S9C, right lower panel).

To confirm increased co-translational ubiquitination (CTU) in DDX3 KO cells, we used an alternative approach that allow us to examine the presence of endogenous ubiquitin conjugates in untransfected cells. Ribosomes collected from WT and DDX3 KO cells were incubated with Bio-Puro and total reaction products were isolated by streptavidin-agarose beads pull-down and immunoblotted with either streptavidin HRP or anti-Ub (FK2) antibody (Figure 6C). Similarly to the HA-Ub conjugated nascent polypeptide results, we detected a broader set of nascent chains modified with ubiquitin in the DDX3 KO as opposed to the WT strain (Figure 6D, right panel). Although there was no significant difference in nascent polypeptides between WT and DDX3 KO cells (Figure 6D, streptavidin-fluorescence panel), much higher levels of these nascent chains were ubiquitinated in cells lacking DDX3 (Figure 6D, anti-Ub panel). Taken together, these results indicate that loss of DDX3 leads to increased ubiquitination of nascent polypeptides, which further supports that ribosomes do not elongate optimally and produce aberrant products that have to be eliminated through CTU and proteasomal degradation.

### Increased co-translational ubiquitination in cells lacking DDX3 correlates with higher recruitment of E3 ubiquitin ligases and proteasome components to the ribosome

In yeast and mammals, co-translational ubiquitination (CTU) is carried out by a complex network of E3 ubiquitin ligases that recognize and polyubiquitylate nascent peptides on stalled ribosomes (4–6,58). Interestingly, LFQ proteomic analysis of ribosomes collected from WT and DDX3 KO cells identified several components of the ubiquitination machinery that were preferentially recruited to DDX3 KO ribosomes. These include E3 ubiquitin ligases of the HECT (Homologous to E6AP C-Terminus) family (59) (LINF_350029800) and the cyclosome APC/C large multi-subunit (LINF_050009100/APC3, LINF_300012300/APC6, LINF_040009000/APC10) catalyzing ubiquitination and degradation of key cell cycle regulatory proteins (60), a ubiquitin-conjugating enzyme E2 (LINF_040011900), the proteasome ubiquitin receptor Rpn13 (LINF_350022600) and the ubiquitin carboxyl-terminal hydrolase UCH37 (LINF_240009000) (Table 2, Supplementary Table S5). LINF_350029800 harbors the characteristic C-terminal HECT domain but has no structured N-terminal domain harboring Armadillo repeats, thus belonging to the ‘Other’ HECT ligase members (59). The anaphase promoting complex (cyclosome) APC3 and APC6 scaffolding subunits are shown to interact with APC10, a core subunit with a vital function in substrate recognition (60). The ubiquitin receptor of the 26S proteasome regulatory subunit Rpn13 facilitates substrate delivery to the proteasome (61). The deubiquitinating enzyme UCH37 binds the 19S proteasome regulatory particle through its C-terminal domain which is recognized by Rpn13 (62) and acts as a debranching deubiquitinase important for promoting proteasomal degradation (63). Another deubiquitinating enzyme (LINF_160012700) was found to be upregulated in DDX3 KO cells as determined by LFQ proteomic analysis of total lysates (Table 1, Supplementary Table S3; not detected in the ribosomal proteome). This protein belongs to the peptidase C19 ubiquitin carboxyl-terminal hydrolase family that regulates cellular Ub levels by removing Ub from the target proteins or breaking up polyubiquitin chains into free Ub monomers. Higher recruitment of components of the ubiquitination machinery to DDX3 KO ribosomes was not due to an increase in their expression as determined by LFQ analysis of the total proteome (Supplementary Tables S3 and S4). Altogether, these results are in line with increased CTU in cells lacking DDX3.

### DDX3 loss leads to the accumulation of cytoplasmic protein aggregates

**Figure 7.**
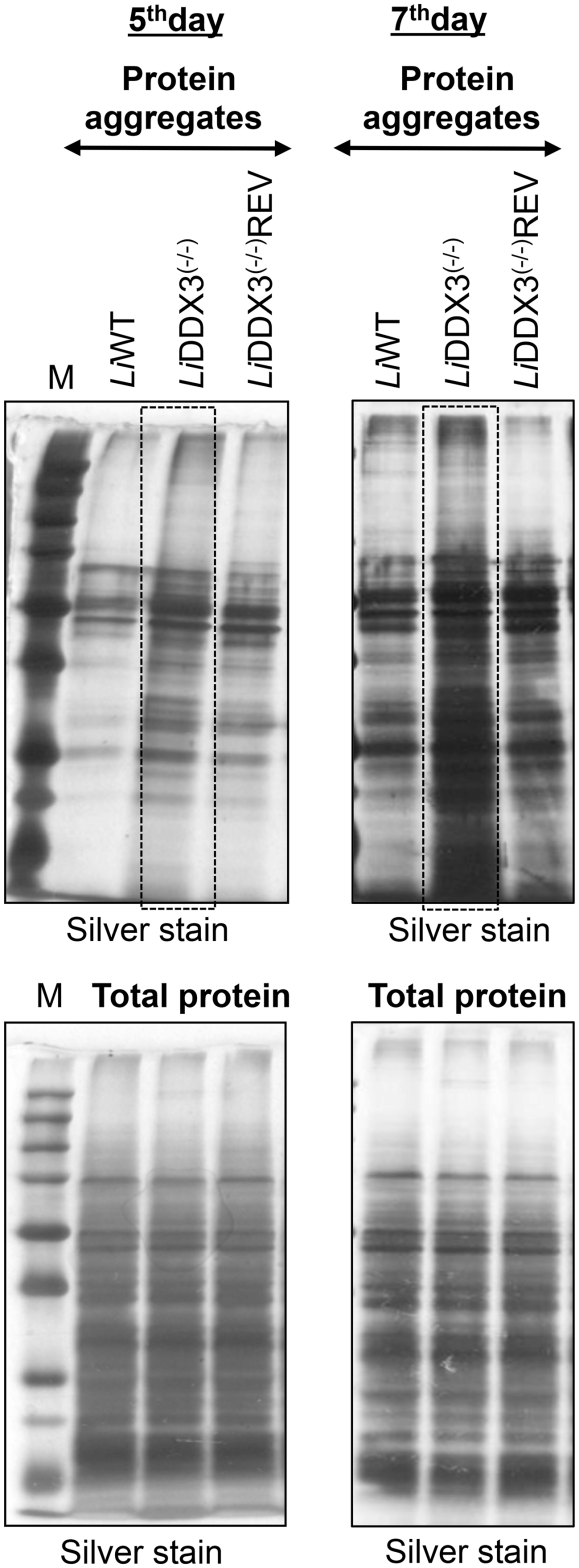
DDX3 loss leads to increased accumulation of detergent-resistant cytoplasmic aggregates. The insoluble protein aggregates were isolated from equal amounts of *L. infantum Li*WT, *Li*DDX3^(−/−)^ and *Li*DDX3^(−/−)^REV lysates as described in Materials and Methods. The aggregate species from 5-day and 7-day stationary-phase mutant parasites lacking DDX3 were resolved on 12% SDS-PAGE gel and detected by silver staining. Total protein silver-stained extracts demonstrate equal protein loading. M: protein ladder. Representative data from one out of five independent experiments with similar results are shown (see also Supplementary Figure S10).

During protein synthesis, nascent polypeptides emerge from the ribosome to fold into functional proteins. Truncated or misfolded newly synthesized polypeptides can form aggregates that must be co-translationally ubiquitinated and rapidly degraded through the ubiquitin-proteasome system to circumvent their deleterious effects for cells (64). Considering that DDX3 loss leads to impaired elongation of translating ribosomes which could possibly produce aberrant polypeptides, we next investigated whether aggregate species could be formed in DDX3 KO cells. For this, we extracted the insoluble (detergent-resistant) protein fraction from equal amounts of total lysates of WT, DDX3 KO and rescue mutant strains (Figure 7) by successive centrifugation and lysis steps, as described before (34) and detailed in Materials and Methods. A silver stained SDS-PAGE gel revealed higher levels of detergent-resistant aggregate species in DDX3 KO cells in comparison to the control strains (Figure 7, Supplementary Figure S10). Aggregate species were gradually accumulating with time as parasites entered their late-stationary phase (Figure 7, Supplementary Figure S10B; days 5 and 7), suggesting that impaired translation was not fully rescued by quality control pathways in cells lacking DDX3.

Interestingly, LFQ proteomic analysis revealed that LINF_330016300, a member of the DnaJ/Hsp40 family of co-chaperones, was preferentially recruited to ribosomes of DDX3 KO cells (Table 2 and Supplementary Table S5). DnaJ/Hsp40 molecular chaperones through their J-domain bind to Hsp70 members and promote their ATPase activity, thus generating the ADP-bound Hsp70 which stably interacts with client proteins to allow their folding or disaggregation (65). Thus, higher ribosomal recruitment of DnaJ/Hsp40 corroborates increased aggregate formation in cells lacking DDX3.

### Increasing HSP70 availability in cells lacking DDX3 partially restores translation

Increased CTU of nascent chains and accumulation of cytosolic aggregates in cells lacking DDX3 implies that arrested polypeptides on stalled ribosomes may be truncated and misfolded. It is well known that ATP-dependent ribosome-associated chaperones such as Hsp70 and its J-domain co-chaperones assist the folding and unfolding of nascent polypeptides as they exit the ribosomal tunnel (66). We have shown previously that *Li*DDX3 co-immunoprecipitates members of the Hsp70 subfamily and DnaJ proteins (23) (see also Supplementary Table S2). Using sucrose gradient fractionation and western blotting of TCA-precipitated proteins from *L. infantum* WT and DDX3 KO fractions with anti-Hsp70 specific antibody we confirmed ribosomal association of the cytoplasmic Hsp70 (cytHsp70) protein (Supplementary Figure S11), as reported before in other eukaryotes.

**Figure 8.**
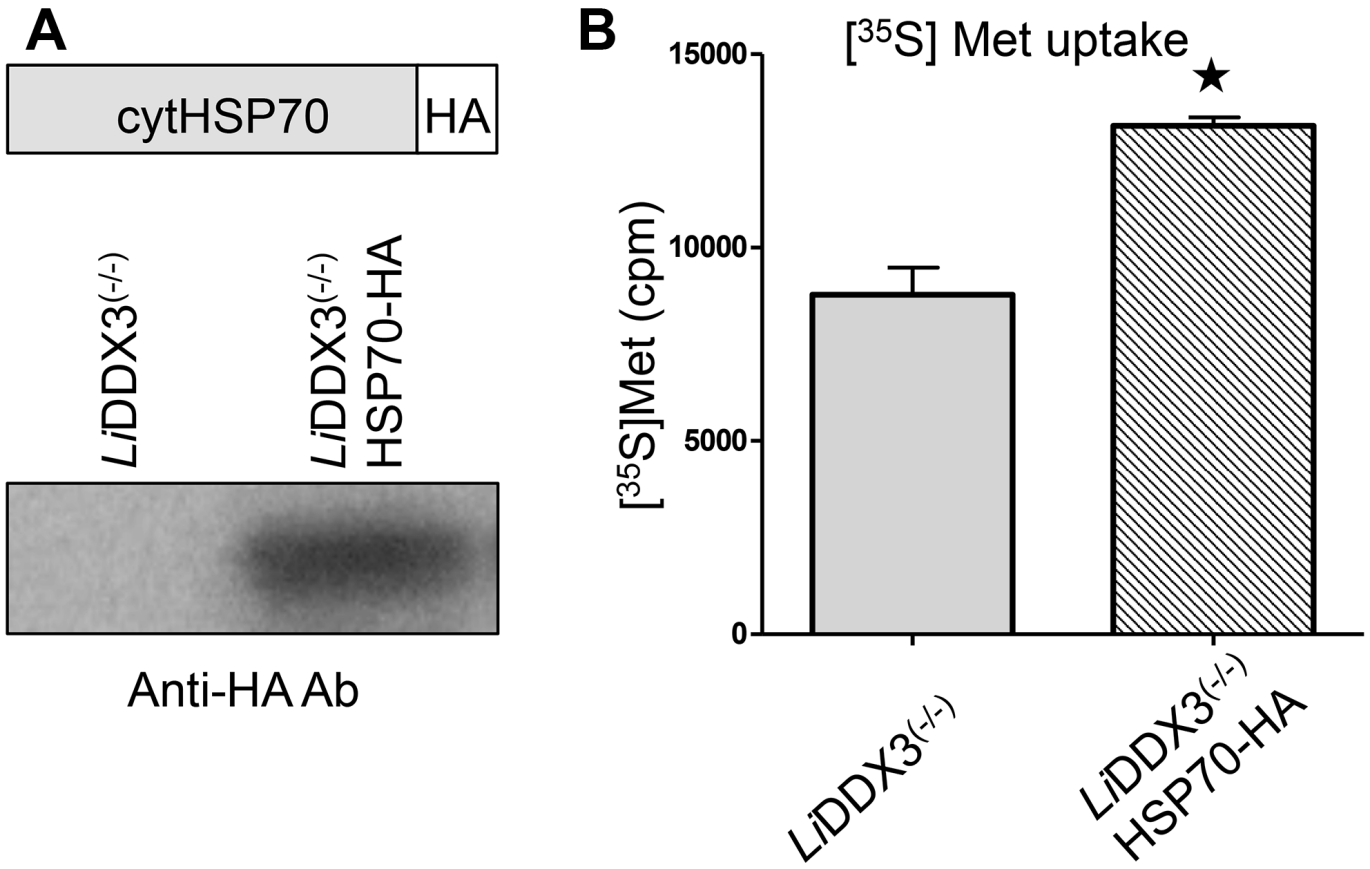
Increasing Hsp70 availability in cells lacking DDX3 partially restores translation rates. (**A**) Schematic illustration of the Hsp70-HA cassette used for overexpressing the cytoplasmic (cyt) Hsp70 protein into the DDX3 knockout mutant (upper panel). Western blot with anti-HA antibody to detect cytHsp70 expression in *Li*DDX3^(−/−)^ cells (lower panel). (**B**) Ectopic overexpression of cytHsp70 into cells lacking DDX3 and its association with ribosomes (see Supplementary Figure S11) improves translation rates as determined by [^35^S] Met incorporation studies. Data are plotted as mean ± standard error from three biological replicates. Statistical significance was assessed by two-tailed unpaired *t*-test. *P< 0.05.

Next, we tested whether overexpressing Hsp70 into DDX3 KO cells could improve translation. Therefore, we engineered a DDX3 KO strain episomally expressing HA-tagged cytHsp70 (LINF_280035000) protein as confirmed by western blotting with anti-HA antibody (Figure 8A). [^35^S]-Met incorporation indicated a ∼1.6-fold increase in *de novo* protein synthesis in DDX3 KO cells overexpressing Hsp70-HA (Figure 8B). Hsp70 overexpression seems also to slightly diminish aggregate formation (Supplementary Figure S10B, day 7).

## DISCUSSION

Several previous reports in yeast and mammalian cells have shown the implication of the multifunctional DEAD-box RNA helicase Ded1/DDX3 in translation initiation (13,17–19,21). However, little is currently known about the role of this protein in post-initiation phases of translation. In this study, we provide evidence supporting that the *Leishmania* DDX3 ortholog is an important contributor to optimal elongation of translating ribosomes by preventing prolonged translation stalls and stimulating dissociation and recycling of arrested ribosomes. We show that translation elongation is impaired upon DDX3 loss with ribosomes accumulating and stalling early in elongation and engaging less in the process, thus reducing the cell's capacity for protein synthesis. Genetic depletion of DDX3 slows down the speed of elongating ribosomes and reduces the recruitment of essential translation termination and recycling factors to ribosomes, which may lead to inefficient dissociation and recycling of elongation-stalled ribosomes. Furthermore, we show that prolonged ribosome stalling in cells lacking DDX3 induces quality control pathways to rescue arrested ribosomes as evidenced by the significant increase in co-translational ubiquitination of nascent polypeptides and the higher recruitment of E3 ubiquitin ligases and components of the proteasome to ribosomes of DDX3 knockout cells. Impaired elongation of translating ribosomes also results in the accumulation of cytoplasmic protein aggregates, which implies that defects in translation overwhelm the normal quality controls.

In recent years, translation elongation has emerged as an important contributor to the regulation of gene expression undergoing multiple quality control checkpoints (2,3). Ribosome speed during elongation is regulated to allow proper folding of the nascent peptides and functional proteins. Prolonged pausing or stalling of translating ribosomes can be detrimental to cells. Thus, cells have evolved quality control mechanisms that recognize defects in translation elongation to recycle arrested ribosomes and degrade aberrant polypeptides and mRNAs (5,7,67,68). Ribosome arrest may happen as a consequence of highly structured or aberrant mRNAs, truncated/misfolded polypeptides, insufficient availability of charged tRNAs, damaged ribosomes, or exogenous stress factors (5,67,68). In *Leishmania* cells lacking DDX3, stalling of elongating ribosomes could possibly be attributed to a combination of factors. It may be caused by the formation of substantial secondary structures in mRNAs. As an ATP-dependent RNA helicase and similarly to its Ded1p yeast ortholog (19,21), DDX3 may disrupt local RNA secondary structures within 5′UTRs or 3′UTRs of mRNA targets. We have reported previously that inactivation of DDX3 leads to increased degradation of the ribosomal RNA, which is exacerbated upon stress and drug-induced cell death (27). Ribosomal RNA degradation could alter ribosome homeostasis and result in ribosome stalling. Alternatively, DDX3 may be needed for displacing protein complexes that impede elongation, hence allowing efficient ribosome translocation. Our data that DDX3 loss slows down the speed of translating ribosomes and results in elongation stalls are in line with this possibility. Also, our results that Rli1/ABCE1 and translational GTPases eRF3 and GTPBP1 are less recruited to ribosomes in the absence of DDX3 support that arrested ribosomes may be inefficiently dissociated and recycled.

Many studies in yeast and mammalian cells strongly support the crucial role Rli1/ABCE1, eRF3 and GTPBP1 play in translation elongation, termination and recycling but also in ribosome quality control (RQC) pathways. In eukaryotes, a stop codon in the ribosomal A site is decoded by a ternary complex consisting of release factors eRF1 and GTP-bound eRF3. After GTP hydrolysis, eRF3 dissociates and the Rli1/ABCE1 ATPase can bind to eRF1-loaded ribosomes to stimulate peptide release and ribosomal subunit dissociation (43,48,49,69,70). Also, eRF1 and eRF3 contribute to co-translational protein quality control (71) and eRF3 was shown to resolve prematurely terminating ribosomes stalled on polylysine segments (72). Rli1/ABCE1 is an essential recycling factor promoting 80S splitting into 40S and 60S subunits after canonical termination (43,48) but is also involved in quality control mRNA surveillance processes on stalled ribosomes (44,46,47,73). In coordination with the eRF1 and eRF3 paralogs Dom34/Pelota and Hbs1/Hbs1L, Rli1/ABCE1 rescues arrested eukaryotic ribosomes on truncated mRNAs to initiate their recycling and the degradation of aberrant mRNAs and polypeptides (44,46,47,73,74). Knockdown or natural loss of Rli1/ABCE1 was shown previously to cause stalling of ribosomes at stop codons and in 3′UTRs of most mRNAs (75,76). Reduced recruitment of ABCE1 to ribosomes of cells lacking DDX3 could similarly lead to stalling of terminating ribosomes or prevent dissociation and recycling of elongation-stalled ribosomes. Moreover, less ribosomal recruitment of eRF3 and ABCE1 could alter either the assembly of eRF1-eRF3 pre-termination complex or the termination/pre-recycling complex containing eRF1-ABCE1, leading to premature termination. Compared to other eukaryotes (74), the *Leishmania* Rli1/ABCE1 ortholog does not seem to interact or associate with Dom34 as suggested by pull-down and proteomic studies. Interestingly, *Leishmania* does not code for Hbs1, and its closest homolog is eRF3 which infers that Hbs1′s function may be fulfilled by eRF3. By contrast to mammalian cells where ABCE1 loss was shown to enhance ribosome rescue by Dom34/Hbs1 (76), our quantitative proteomic analysis did not reveal any functional compensation by Dom34 for the decreased recruitment of ABCE1 to DDX3 KO ribosomes. These data suggest that ribosome stalling in the absence of DDX3 may be not depend on Dom34/Hbs1-mediated ribosome rescue. GTPBP1 is an unconventional translational GTPase most closely related to eRF3 and Hbs1 that in addition to its elongation activity plays important roles in mRNA surveillance and RQC (50,51) and it was also shown to resolve paused ribosomes (77). Thus, based on previous reports in other eukaryotes, decreased recruitment of ABCE1, eRF3 and GTPBP1 to ribosomes of *Leishmania* cells lacking DDX3 could contribute to the accumulation of elongation-stalled ribosomes or termination-stalled ribosomes and or to prematurely terminating ribosomes. Although our data clearly indicate defects in post-initiation steps of translation in the absence of DDX3, we cannot rule out the possibility that translation initiation is affected as a consequence of reduced ribosome recycling. Several reports have proposed that ABCE1 somehow orchestrates translation at the crossroad between recycling and initiation by altering initiation complex assembly (78). Our polysome analysis data indicating much less association of ABCE1 with the 40S subunit (co-sedimenting with the 43S pre-initiation complex) in cells lacking DDX3 are in line with this possibility.

Why Rli1/ABCE1, eRF3 and GTPBP1 are less recruited to ribosomes in the absence of DDX3 albeit they are similarly expressed between WT and DDX3 KO cells remains to be elucidated. Polysome analysis and pull-downs of ribosomal proteins, DDX3 and Rli1 combined to LC-MS/MS studies suggest ribosomal association of DDX3 and its interaction (direct or indirect) with Rli1 and the release factors eRF1 and eRF3. Recent data in *S. cerevisiae* have shown a direct interaction of Rli1 with eRF3 (70). It has also been shown that Rli1 requires eRF3 for binding to 80S complexes (43) and it functions in concert with eRF1 to stimulate peptide release and ribosomal subunit dissociation (43,48,69). DDX3 may stimulate/stabilize interactions between Rli1 and eRF1/eRF3 or Rli1 and ribosome rescue factors or it may induce stronger binding of Rli1 and release factors to the ribosome or it may participate in the assembly of termination/pre-recycling and ribosome rescue complexes, hence facilitating peptide release, ribosome dissociation and recycling upon translation arrest or during canonical termination. In comparison, the human RNA helicase DDX19 (Dbp2 in yeast) was shown to stabilize ribosomal elongation and termination complexes in the presence of release factors (79).

Furthermore, we show that perturbed translation elongation upon DDX3 loss triggers quality-control mechanisms, leading to increased co-translational ubiquitination (CTU) of nascent chains. CTU is part of the RQC process and rescues prolonged ribosome stalling by polyubiquitylating truncated/aberrant nascent polypeptides through the action of a complex network of E3 ubiquitin ligases for proteasomal degradation (58,68,80). *Leishmania*, similarly to other eukaryotes (32,80), displays low levels of CTU within active translation complexes but these are significantly increased upon DDX3 loss as determined by biotinylated-puromycin labeling assays. Consistent with the increased CTU, we show that cells lacking DDX3 preferentially recruit to the ribosome a member of the HECT family of E3 ubiquitin ligases and subunits of the E3 ubiquitin ligase anaphase promoting complex (APC/C or cyclosome), which catalyzes the ubiquitination of key cell cycle regulatory proteins (60). These E3 ligases may be contributing to increased CTU in DDX3 KO cells but further studies will be necessary to assess their direct role in this process. Preliminary studies indicated a cell cycle defect in DDX3 KO cells (Padmanabhan, unpublished), which may correlate with higher recruitment of the APC/C complex to the ribosome. Increased CTU in cells lacking DDX3 implies that ribosomes are not elongating optimally, which corroborates our data indicating slower movement and stalling of translating ribosomes and reduced rate of polypeptide synthesis. Although ribosome stalling early in the elongation process seems to be for the most part reversible as suggested by harringtonine treatment, activation of CTU implies that stalls should be prolonged requiring resolution by ribosome rescue and quality control pathways, as previously described in other eukaryotes (80,81). Our results indicating higher levels of Ub-modified proteins within the 60S subunit in DDX3 KO cells are in line with previous reports that 60S-nascent chain complexes are recognized by the RQC machinery to allow nascent chain polyubiquitination and subsequent degradation by the proteasome (5,6,58,67). As K48-linked chains are the predominant linkage type in cells (>50%) and their role is to target proteins to the proteasome for degradation (54), it is likely that a significant part of the anti-Ub (K29-, K48- and K63-linked chains) signal detected on DDX3 KO ribosomes corresponds to nascent chains accumulating on stalled ribosomes in the absence of DDX3, as also reinforced by our Bio-Puro tagging assays. Our data also support increased ubiquitination of polypeptides associated with the ribosomes in the absence of DDX3. Indeed, we observed ubiquitination of 60S/40S associated polypeptides enriched by pull-down assays and higher levels of ubiquitination on intact ribosomes collected from DDX3 KO cells which may include ribosomal proteins or factors with regulatory roles in ribosome/translation processes as reported previously in yeast and mammals under conditions of ribosome stalling and impaired translation (55). Ubiquitin is known to play a key regulatory role in several ribosome processes, including quality control of arrested peptides and faulty mRNA, and modulation of translation in response to stress (82).

While CTU of nascent polypeptides on elongation-stalled ribosomes is increased in cells lacking DDX3, we show an accumulation of cytoplasmic protein aggregates which implies that impaired translation elongation overwhelms the normal quality controls. Aberrant nascent polypeptides are co-translationally ubiquitinated and transferred via the AAA + ATPase VCP/Cdc48 and its cofactors to the proteasome for degradation to avoid accumulation of toxic aggregation products (66,83). It was shown previously that if ubiquitination of stalled nascent chains is compromised, these polypeptides form aggregates in the cytosol or within organelles (84). To handle aggregate formation, cells often upregulate molecular chaperones to allow protein refolding (66). The partial recovery of translation by overexpressing Hsp70 into *Leishmania* DDX3 KO cells supports the production of aberrant/misfolded polypeptides which cannot be completely rescued by quality control pathways. Previous studies in mammals have shown that translation elongation defect due to proteotoxic stress (53) or to heat shock (41,53) can partially be rescued by overexpressing Hsp70. Hsp70 is the major eukaryotic ribosome-associated chaperone and the first reported to bind co-translationally to nascent chains together with J-domain co-chaperones triggering ATP hydrolysis to facilitate protein-folding processes and (re)establish protein homeostasis (52). Interestingly, here, we show that a member of the DnaJ/Hsp40 family was preferentially recruited to ribosomes in cells lacking DDX3, which is in line with the formation of aggregates.

In summary, these data highlight a new role of DDX3 in post-initiation steps of translation. We provide evidence that the *Leishmania* DDX3 ortholog is an important contributor to optimal elongation of translating ribosomes by preventing prolonged translation stalls and stimulating dissociation and recycling of arrested ribosomes. Further studies will be necessary however to better understand how DDX3 through different partnerships with elongation and ribosome rescue and recycling factors contributes to these processes.

## DATA AVAILABILITY

The mass spectrometry proteomics data have been deposited to the ProteomeXchange Consortium via the PRIDE (85) partner repository with the dataset identifier PXD020667. Reviewer account details: Username: reviewer72938@ebi.ac.uk; Password: JKikIFNf.

## Supplementary Material

gkab667_Supplemental_Files

## Notes

Present address: Prasad Kottayil Padmanabhan, Galvin Life Sciences Center, Notre Dame University, Notre Dame, IN 46556, USA.

Present address: Filipe Colaço Mariz, Tumorvirus-Specific Vaccination Strategies, Deutsches Krebsforschungszentrum (DKFZ), 69120 Heidelberg, Germany.
