# Supplementary material for: Genetic depletion of the RNA helicase DDX3 leads to impaired elongation of translating ribosomes triggering co-translational quality control of newly synthesized polypeptides
Source: Nucleic Acids Res. 2021 Aug 6;49(16):9459–78. doi: 10.1093/nar/gkab667 (PMC8450092; doi:10.1093/nar/gkab667)
Supplement: gkab667_Supplemental_Files [file gkab667_supplemental_files.zip › Supplementary Data.pdf]

SUPPLEMENTARY DATA

Genetic inactivation of the RNA helicase DDX3 leads to impaired elongation of translating ribosomes triggering co-translational quality control of newly synthesized polypeptides

Prasad Kottayil Padmanabhan[1,2#], Gabriel Reis Ferreira[1,2¶], Ouafa Zghidi-Abouzid[1,2¶], Camila Oliveira[1,2], Carole Dumas[1,2], Filipe Colaço Mariz[1,2¥] and Barbara Papadopoulou[1,2*]

[1]Research Center in Infectious Diseases, Division of Infectious Disease and Immunity CHU de Quebec Research Center-University Laval

[2]Department of Microbiology, Infectious Disease and Immunology, Faculty of Medicine, University Laval, Quebec, QC. Canada G1V 4G2

[¶]Equal contribution

[#]Current address: Galvin Life Sciences Center, Notre Dame University, Notre Dame, IN 46556, USA

[¥]Current address: Tumorvirus-Specific Vaccination Strategies, Deutsches Krebsforschungszentrum (DKFZ), 69120 Heidelberg, Germany

* Corresponding author: Prof. Barbara Papadopoulou

Research Center of the CHU de Québec (CHUL)-Université Laval

2705 Laurier Blvd., Quebec (QC), Canada G1V 4G2

Phone: 418-525 4444, ext. 47608

Email: barbara.papadopoulou@crchudequebec.ulaval.ca

Running title: DDX3 regulates elongation of translating ribosomes

Key words: *Leishmania*; DDX3; Ribosome stalling; Translation elongation; Ribosome recycling; ABCE1/Rli1; eRF3; Co-translational ubiquitination; Protein aggregates; Polysome analysis; Label-free quantitative proteomics; Biotinylated-puromycin labeling

Supplementary Figure Legends

Supplementary Figure S1. Sequence alignment of the *L. infantum* (Leish) DEAD-box RNA helicase DDX3 with the *Saccharomyces cerevisiae* (Sc) Ded1 (NCBI, CAA99419) and the *Homo sapiens* (Hs) DDX3X (NCBI, NP_001347.3) orthologous proteins. The ClustalW multiple alignment using Bioedit sequence Alignment Editor was applied here. Gaps denoted by dashes have been introduced into the output by ClustalW in order to align the sequences. Identical amino acids conserved in all three proteins are indicated by an asterisk. The 12 conserved motifs of the DEAD-box RNA helicase core, as well as conserved residues and motifs within the N- and C-terminal extensions that are specific to the Ded1/DDX3 subfamily are indicated by a blue bar. Conserved motifs involved in ATP binding and hydrolysis [(motif Q, Walker A motif (I), LDEADRML or Walker B motif (II) and VI motif] are indicated in blue bold characters. Conserved motifs involved in RNA binding are indicated in red [(motif Ia, glycine duplets GG (motif Ib) not present in all superfamily 2 members, Ic, IV, IVa, and V). The SAT motif (motif III) important for RNA-dependent hydrolysis of ATP (but not the affinity for ATP) and single-stranded RNA binding is highlighted in green. Motifs III and Va have also been suggested to make the link between the ATPase and helicase activities.

Supplementary Figure S2. *Li*DDX3 is mostly localized to the cytosol. Schematic illustration of the *Li*DDX3-GFP fusion protein expressed by *L. infantum* and used for subcellular localization studies (upper panel). To generate the *L. infantum* recombinant strain expressing the *Li*DDX3-GFP protein, the full length *LiDDX3* gene without the stop codon was PCR-amplified using primers described in Supplementary Table S1 and cloned into the HindIII site of the neomycin (NEO) expressing vector pGEM7ZαNEOα-GFP and then transfected into *L. infantum* by electroporation. The green fluorescence signal (DDX3) was observed in the cytoplasm of *L. infantum* promastigotes (lower panel). Hoechst 33258 (blue stain) specifically stains DNA of nucleus and kinetoplast. The green fluorescence signal did not merge with that of the Hoechst 33258, suggesting only a cytoplasmic localization for *Li*DDX3-GFP protein.

Supplementary Figure S3. *De novo* protein synthesis is decreased in cells lacking DDX3. The same number of cells (10^7 cells) from exponentially grown *L. infantum* WT (*Li*WT), *Li*DDX3$^{(-/-)}$ (DDX3 KO mutant) and *Li*DDX3$^{(-/-)}$REV (add-back mutant) after [^{35}S] Met uptake at various time points (from 2.5-

30 min) was lysed and resolved in 12% SDS-PAGE. The gel was then transferred to Immobilon-P polyvinylidene difluoride (PVDF) membrane and exposed to a photographic film (upper panel) to visualize [^{35}S] Met labeled proteins over the 30 min period for each strain. These data clearly indicate that much less proteins are synthesized in cells lacking DDX3 in comparison to the WT and rescue controls. The same membrane was stained with Coomassie Blue (CBB) to show the amount of proteins loaded in each lane (lower panel). Although the amounts of total protein loaded are not equal at each time point between the three strains, no important variations were observed, hence suggesting that steady-state levels do not significantly differ between *Li*WT, *Li*DDX3 knockout and rescue strains. While a slight decrease in total protein (especially for the 2.5 min time point) was observed in the DDX3 KO strain compared to the controls, the *de novo* ^{35}S signal was substantially reduced in cells lacking DDX3. Actually, at the 30 min time point there is more protein in the DDX3 knockout strain as determined by CBB staining compared to that of WT, still there is less amount of *de novo* protein synthesis. Representative data of three independent experiments are shown here.

Supplementary Figure S4. Polysome analysis by density gradient fractionation in the presence of the translation inhibitors cycloheximide or harringtonine. Cytoplasmic lysates from *L. infantum* WT (*Li*WT) and DDX3 knockout (*Li*DDX3$^{(-/-)}$) cells (RNA equivalent of 600 µg) were layered on top of a linear 15%-45% sucrose gradient and sedimented following ultracentrifugation to allow the separation of free ribonucleoprotein complexes (RNP) particles from 40S and 60S ribosomal subunits, 80S monosome and polysomal fractions according to their respective densities. Polysome profiles were generated by continuous absorbance measurement at 254 nm. In the presence of cycloheximide **(**CHX), polysomes were accumulated as opposed to untreated samples (-CHX) (compare upper and middle panels). Harringtonine (HAR) (30 µg/ml; Cayman Chemicals) treatment resulted in the run off of polysomes that was much more pronounced in DDX3 knockout cells. HAR inhibits translation initiation by halting ribosomes at initiation codons while allowing already engaged ribosomes to complete translation through termination. The arrow indicates the 2-ribosome containing fraction in cells lacking DDX3 that was retained following HAR treatment.

Supplementary Figure S5. Parasites lacking DDX3 are more susceptible to the translation elongation inhibitors cycloheximide and puromycin. *Li*WT, *Li*DDX3$^{(-/-)}$ and *Li*DDX3$^{(-/-)}$REV strains were treated with various concentrations of puromycin dihydrochloride (PURO) (0 to 25 µg/ml) (**A**) and cycloheximide (CHX) (0 to 400 ng/ml) (**B**) and cultured at pH 7.0 and 25°C in SDM-79 medium

supplemented with 10% heat-inactivated FCS for 96 h. The drug sensitivity was evaluated by measuring the OD at 600 nm in T25 flasks. These experiments were conducted twice in triplicates with similar results. Error bars indicate the standard deviation of the mean.

Supplementary Figure S6. A DDX3 mutant lacking the LDEADRM motif involved in ATP binding and hydrolysis failed to rescue slowdown of ribosome movement in cells lacking DDX3. The ribosomal half-transit time was measured in *Li*DDX3$^{(-/-)}$ cells rescued with a DDX3 mutant protein lacking the DEAD-box motif (ΔLDEADRM) known to be essential for ATP binding and hydrolysis. The ribosomal half-transit time was determined by measuring the kinetics of [^{35}S]-Met incorporation into total protein (nascent and released polypeptides) in post-mitochondrial supernatant (PMS) and into completed polypeptides released from the ribosome in post-ribosomal supernatant (PRS), as detailed in Materials and Methods. The ribosomal half-transit time (Ts in seconds) was obtained as the displacement in time between the intercepts of the PMS (total CPM) and PRS (released CPM) lines on the time axis, which were determined by linear regression analysis.

Supplementary Figure S7. Ectopic expression of Rli1/ABCE1-HA and eRF3-HA into *L. infantum* wild type and DDX3 knockout cells. Western blot analysis of *L. infantum* wild type (WT) and DDX3 knockout (KO) cells expressing Rli1/ABCE1-HA and/or eRF3-HA with anti-HA antibody indicated similar expression of ABCE1-HA and eRF3-HA between WT and DDX3 KO cells. The anti-alpha tubulin antibody was used as protein loading control. ABCE1-HA and eRF3-HA are stably expressed into WT and DDX3 KO cells as part of episomal vectors, and because plasmid copy number can vary between transfectants, we examined the expression of ABCE1-HA and eRF3-HA proteins by western blotting and found that it was similar between the two strains.

Supplementary Figure S8. 40S and 60S ribosomal protein pull-downs to evaluate the extent of ribosome-associated ubiquitination upon DDX3 inactivation. Ribosomal proteins and ribosome-associated factors were enriched by immunoprecipitation using the 60S ribosomal protein L13a or the 40S ribosomal protein S6 HA-tagged at the C-terminus and independently transfected into *Li*WT and *Li*DDX3$^{(-/-)}$ cells. L13a-HA and S6-HA co-immunoprecipitates were analyzed by western blotting with anti-HA antibody (**A**) and anti-ubiquitin specific antibody (FK2) (**B**). Also shown here is the DDX3 IP from the *Li*DDX3$^{(-/-)}$ strain complemented either with DDX3-HA (add-back mutant) or with the mutant protein DDX3ΔLDEADRM-HA (unable to rescue) followed by western blotting with FK2 Ab.

Supplementary Figure S9. Parasites lacking DDX3 exhibit increased co-translational ubiquitination of nascent polypeptide chains. (**A**) Schematic diagram of 2xHA-ubiquitin (Ub) is shown (upper panel). The 2xHA-Ub expressing plasmid was stably transfected into *L. infantum* (*Li*) WT and DDX3 knockout (*Li*DDX3$^{(-/-)}$) strains, and HA-Ub moieties were efficiently conjugated to *Leishmania* proteins as revealed by western blotting using anti-HA antibody (lower panel). Expression of 2xHA-Ub was higher in WT in comparison to the DDX3 knockout strain due to the 2xHA-Ub plasmid copy number variation between these two strains (see explanations in B). (**B**) The copy number of the 2xHA-Ub expressing plasmid varies between WT and DDX3 knockout strains as determined by Southern blot hybridization. Genomic DNA from *L. infantum* WT and *Li*DDX3$^{(-/-)}$ cells expressing the 2xHA-Ub plasmid was digested with XbaI, resolved on 1% agarose gel, and subjected to Southern blot analysis. The membrane was hybridized with ubiquitin ORF probe specifically recognizing both the endogenous Ub allele (~12 kb) and the 2xHA-Ub plasmid (around 5 kb). Normalization with the endogenous Ub signal revealed that the 2xHA-Ub plasmid copy number was ~4-fold higher in WT in comparison to the DDX3 knockout mutant. (**C**) Schematic diagram of the *in vitro* biotin-puromycin (Bio-Puro) conjugation approach used to enrich for nascent polypeptide chains. Ribosomes from stable *Li*WT and *Li*DDX3$^{(-/-)}$ transfectants expressing a 2xHA-Ub plasmid were collected by ultracentrifugation through 35% sucrose cushion and incubated *in vitro* with Bio-Puro (left panel). Biotin-labeled HA-Ub conjugated nascent polypeptides were isolated by HA-magnetic beads pull-down and visualized with fluorescently tagged streptavidin (streptavidin-HRP antibody) (right panel, top). The same membrane was used for immunoblotting with anti-HA antibody to detect the HA-ubiquitin conjugated polypeptides (both biotin^{+} and biotin^{-}) (right panel, bottom). Representative data of two independent experiments are shown.

Supplementary Figure S10. Cytoplasmic protein aggregates accumulate over time in cells lacking DDX3. The insoluble protein aggregates were isolated from equal quantity of *L. infantum* wild type (*Li*WT), cells lacking DDX3 (*Li*DDX3$^{(-/-)}$) and the add-back mutant (*Li*DDX3$^{(-/-)}$REV) lysates, resolved on 12% SDS-PAGE gel and visualized by silver staining. Total protein extracts stained with silver demonstrate equal protein loading. Four independent experiments are shown in panels (**A)** and **(B)** with parasite cultures of day 5th and day 7th. Also shown *Li*DDX3$^{(-/-)}$ cells ectopically expressing the

cytoplasmic HSP70 epitope-tagged at its C-terminus with HA (HSP70-HA) where aggregate species slightly decreased (**B**, right panel, 7th day).

Supplementary Figure S11. Ribosomal distribution of cytHsp70 in *L. infantum* wild type and DDX3 knockout strains. A 15%-45% sucrose gradient fractionation of *L. infantum* wild type (*Li*WT) and DDX3 knockout cells (*Li*DDX3$^{(-/-)}$) and immunoblotting with cytosolic (cyt) anti-Hsp70 monoclonal antibody showed that cytHsp70 is associated with ribosomal subunits, 80S monosomes, and polysomes. A representative blot of two independent experiments is shown here.

```
Leish DDX3   ---------------------------------MYKNQAQPQPQPQPQPQAQPQEPSAVAG      28
Sc Ded1      ------------MAELSEQVQNLSINDNNEN----G-YV-PPHLRGKPRSARNNSSNYNN      42
Hs DDX3X     MSHVAVENALGLDQQFAGLDLNSSDNQSGGSTASKGRYI-PPHLRN--REATKG--FYDK      55
                                                  :.      *: :    :       . :

Leish DDX3   ---------AMGGYGSYQQQNRQYGGGNRGFNDAQGTYGGGFN-R---NGGGGGFRGGFG      75
Sc Ded1      ---------------NNGGYNGGRGGGSFFSNNRRGGYG---N-----N---GGFFGGNN      75
Hs DDX3X     DSSGWSSSKDKDAYSSFGSRSDSRGKSSFFSDRGSGSRG-RFDDRGRSDYDGIGSRGDRS      114
                            .     .    * ..    :    *   *               . *    *. .
                                 N-terminal extension
Leish DDX3   GFGGGARGPP-REFVGNNHYH------REEKSEEEIFK--EH----TPGINFDQYEAIKV      122
Sc Ded1      GGSRSNGRSGGRWIDGK-HV----PAPRNEKAEIALFGVPEDPNFQSSGINFDNYDDIPV      130
Hs DDX3X     GFGKFERGGNSRWCDKSDEDDWSKPLPPSERLEQELFSGG---N--T-GINFEKYDDIPV      168
             * .     .     *: .          .   : *   *    :*          . ****: *: * *
                                                   Q motif             motif I
Leish DDX3   SITPNDV-EPAESFATMALAPALAENVNRCRYQKPTPVQKYGIPCVLNGSDLMACAQTGS      181
Sc Ded1      DASGKDVPEPITEFTSPPLDGLLLENIKLARFTKPTPVQKYSVPIVANGRDLMACAQTGS      190
Hs DDX3X     EATGNNCPPHIESFSDVEMGEIIMGNIELTRYTRPTPVQKHAIPIIKEKRDLMACAQTGS      228
             . : ::       .*     :    :    *::    *: :******:.:* : :   **********
                                                              motif Ia
Leish DDX3   GKTAAYLIPAINFMLVNNLNRA--------KPTNSQSAPSALVLSPTRELSIQIYEEGRK      233
Sc Ded1      GKTGGFLFPVLSESFKTGPSPQP---ESQGSFYQRKAYPTAVIMAPTRELATQIFDEAKK      247
Hs DDX3X     GKTAAFLLPILSQIYSDGPGEALRAMKENGRYGRRKQYPISLVLAPTRELAVQIYEEARK      288
             ***..:*:* :.          . .                  . :   *  :::::*****: **::*.:*
                      motif Ib                 motif Ic               motif II
Leish DDX3   FTYRTGIRCVVVYGGADPRHQIHELTRGCGLLVATPGRLSDMFTRGYTRYSDVRFLVLDE      293
Sc Ded1      FTYRSWVKACVVYGGSPIGNQLREIERGCDLLVATPGRLNDLLERGKISLANVKYLVLDE      307
Hs DDX3X     FSYRSRVRPCVVYGGADIGQQIRDLERGCHLLVATPGRLVDMMERGKIGLDFCKYLVLDE      348
             *:**: ::    *****:    :*:::: *** ********* *:: **          ::*****
DEAD                                          motif III
Leish DDX3   ADRMLDMGFEPQIRAIVQGPDSDMPPPGERQTLLYSATFPKEIQQMAREFLYHHHFLQVG      353
Sc Ded1      ADRMLDMGFEPQIRHIVE--DCDMTPVGERQTLMFSATFPADIQHLARDFLSDYIFLSVG      365
Hs DDX3X     ADRMLDMGFEPQIRRIVE-QD-TMPPKGVRHTMMFSATFPKEIQMLARDFLDEYIFLAVG      406
             ************** **:   *   *  *  * *:*:::***** :** :**:** .: ** **
                                                     motif IV
Leish DDX3   RVGSTTENITQDVRWVEDMDKRGCLLEVLKEH-QGERVLVFVEKKRDADYLERYLRQSRI      412
Sc Ded1      RVGSTSENITQKVLYVENQDKKSALLDLLSAS-TDGLTLIFVETKRMADQLTDFLIMQNF      424
Hs DDX3X     RVGSTSENITQKVVWVEESDKRSFLLDLLNATGKDSLTLVFVETKKGADSLEDFLYHEGY      466
             *****:*****.* :**: **:. **::*.      .   .*:***.*: ** *   :*   .
                motif IVa                motif V motif Va
Leish DDX3   PCSSIHGDRVQREREEALDIFKSGVCRVLVATDVASRGLDIPNVAVVVQYDLPSNIDDYV      472
Sc Ded1      RATAIHGDRTQSERERALAAFRSGAATLLVATAVAARGLDIPNVTHVINYDLPSDVDDYV      484
Hs DDX3X     ACTSIHGDRSQRDREEALHQFRSGKSPILVATAVAARGLDISNVKHVINFDLPSDIEEYV      526
              .::***** * :**.**   *:**  . :**** **:***** **   *:::****:::**
motif VI
Leish DDX3   HRIGRTGRAGKRGTAISFFNDKNRNIVDDLIPLLRETNQTVLPEVQALAKKPNVQNQPRG      532
Sc Ded1      HRIGRTGRAGNTGLATAFFNSENSNIVKGLHEILTEANQEVPSFLKD-AMMSA----P-G      538
Hs DDX3X     HRIGRTGRVGNLGLATSFFNERNINITKDLLDLLVEAKQEVPSWLENMAYEHHYKGSSRG      586
             ********.*: * * :***   * **...*   :* *::* *    ::  *          *
    C-terminal extension
Leish DDX3   RGRGGFRSGGFGGGFGGGYGGGYGGRGGYRGGGGFGGGYGGGYGGGYGGNNGGYRPRGGY      592
Sc Ded1      -SRSNSRRGGFGRNNN-----------RDYRKAGGASAGGWGSSR---SRDNSF-R-----      578
Hs DDX3X     RSKSSRFSGGFGA-------------RDYRQSSGASSSSFSSSRASSSRSGGGGHGSSRG      634
              .:..    ****                  .**   .*    .    ..:     .. ..   .

Leish DDX3   -GGGGYGG-----GYGGDRNMR--SDVFGQ      614
Sc Ded1      -GGSGWGSDSKSSGWG-NSGGSNNSSWW--      604
Hs DDX3X     FGGGGYGGFYNSDGYGGNYN-SQGVDWWGN      662
              **.*:*.      *:*.. .    . :
```

Fig. S1

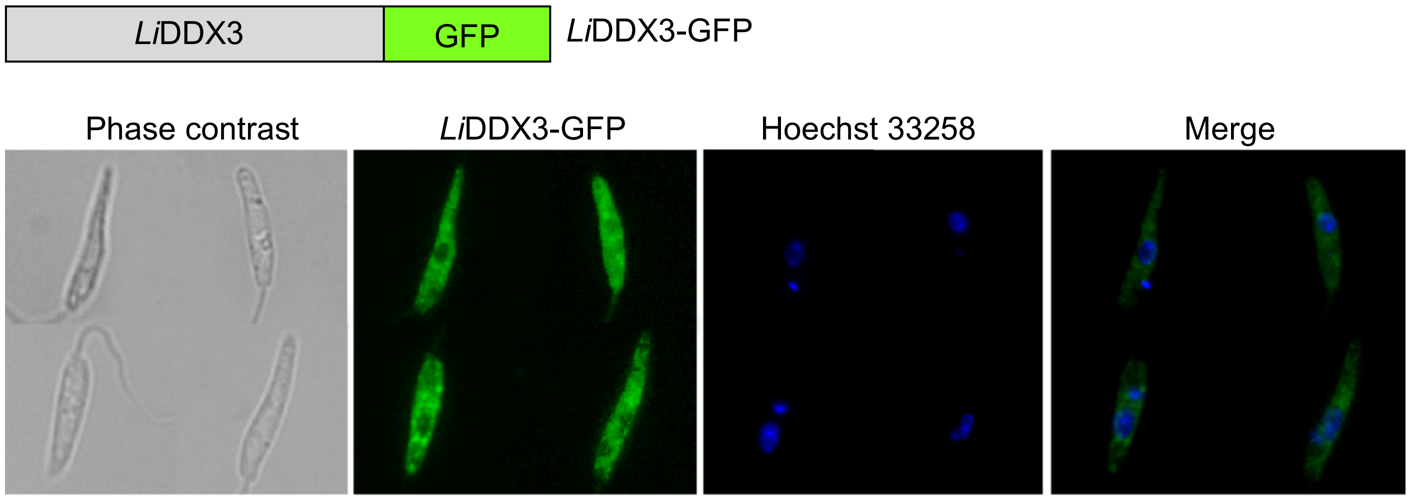

*Li*DDX3
GFP
*Li*DDX3-GFP
Phase contrast
*Li*DDX3-GFP
Hoechst 33258
Merge

Fig. S2

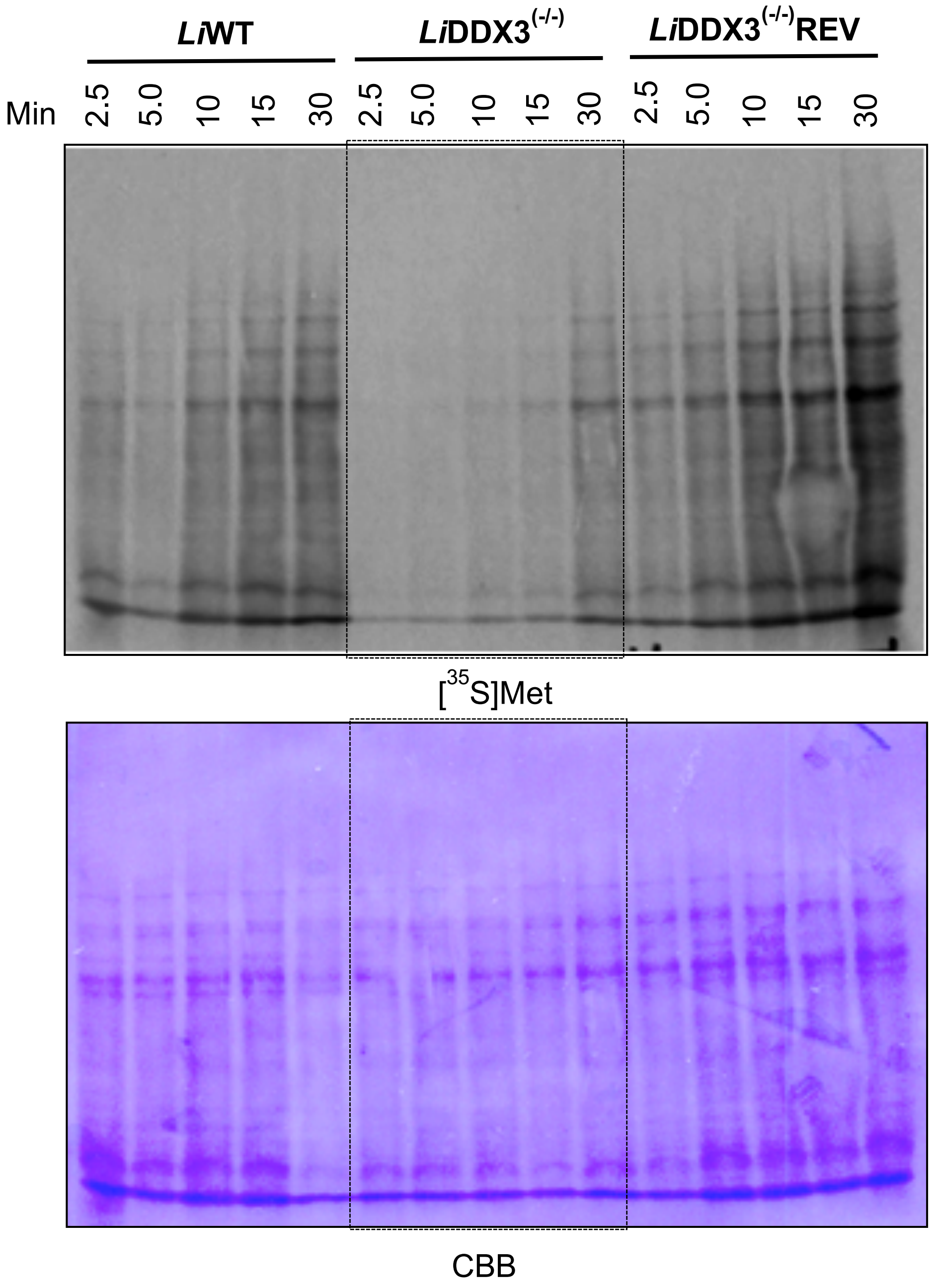

LiWT
LiDDX3(-/-)
LiDDX3(-/-)REV
Min
2.5 5.0 10 15 30 2.5 5.0 10 15 30 2.5 5.0 10 15 30
[35S]Met
CBB

Fig. S3

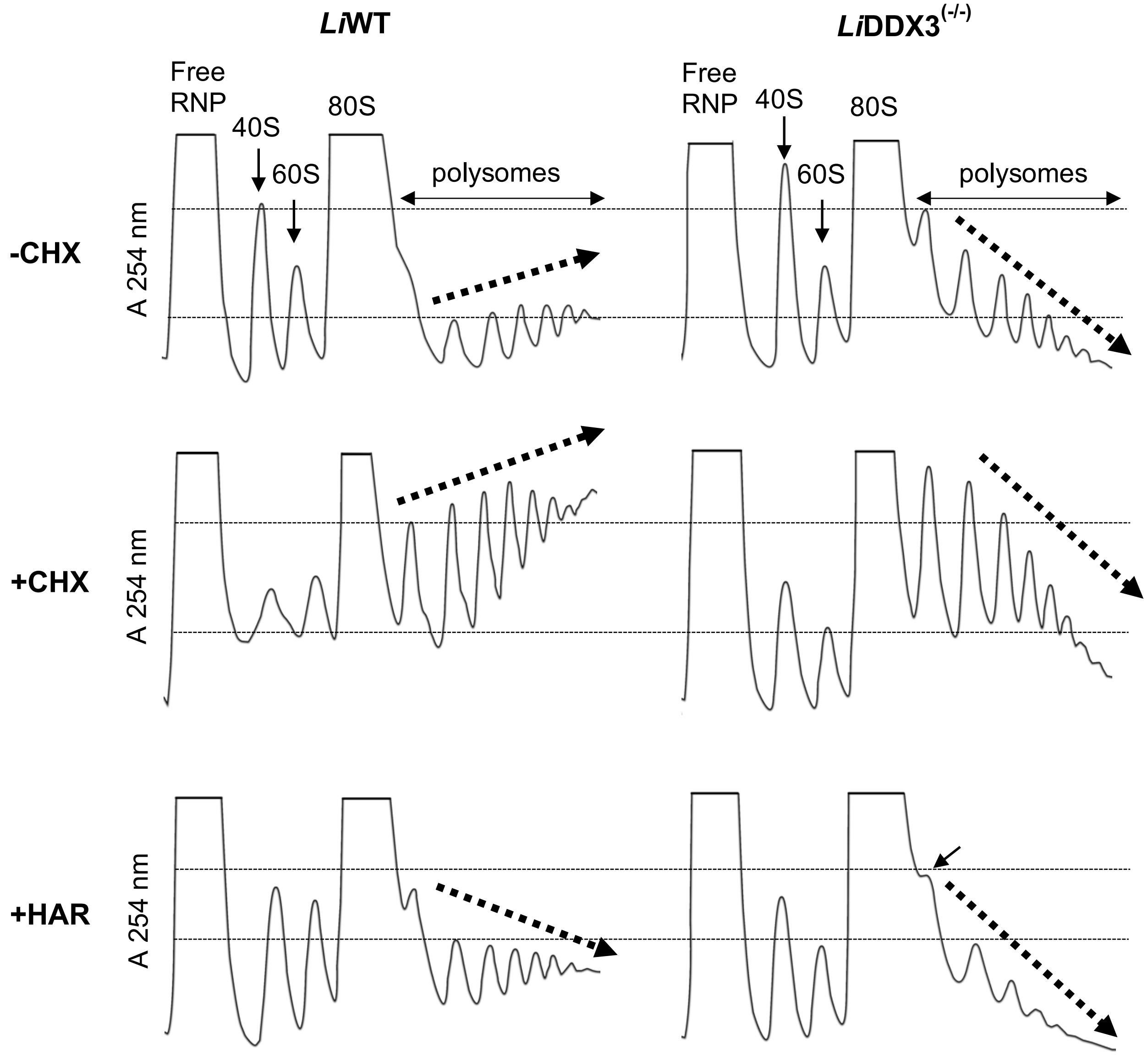

LiWT
LiDDX3(-/-)
Free RNP
40S
60S
80S
polysomes
Free RNP
40S
60S
80S
polysomes
-CHX
+CHX
+HAR
A 254 nm
A 254 nm
A 254 nm

Fig. S4

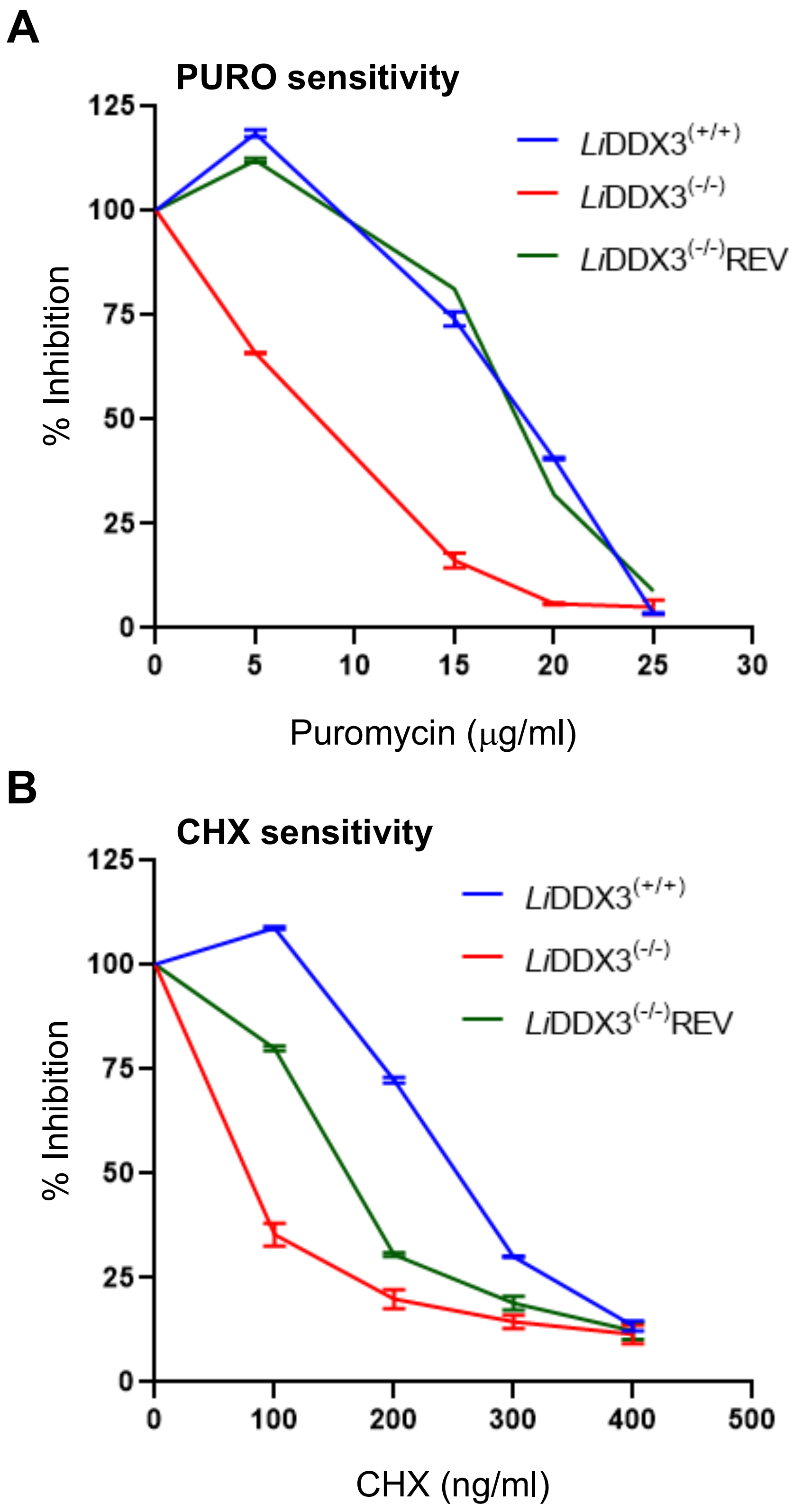
A
PURO sensitivity
125
100
75
50
25
0
% Inhibition
0
5
10
15
20
25
30
Puromycin (μg/ml)
LiDDX3(+/+)
LiDDX3(-/-)
LiDDX3(-/-)REV
B
CHX sensitivity
125
100
75
50
25
0
% Inhibition
0
100
200
300
400
500
CHX (ng/ml)
LiDDX3(+/+)
LiDDX3(-/-)
LiDDX3(-/-)REV

Fig. S5

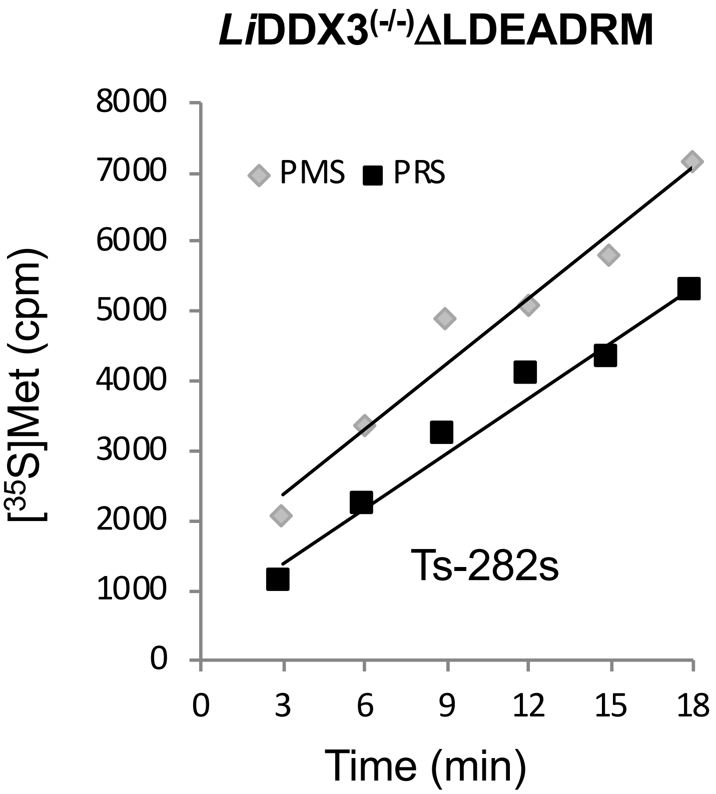
*Li*DDX3$^{(-/-)}$ΔLDEADRM
8000
7000
6000
5000
4000
3000
2000
1000
0
PMS
PRS
Ts-282s
[^{35}S]Met (cpm)
0
3
6
9
12
15
18
Time (min)

Fig. S6

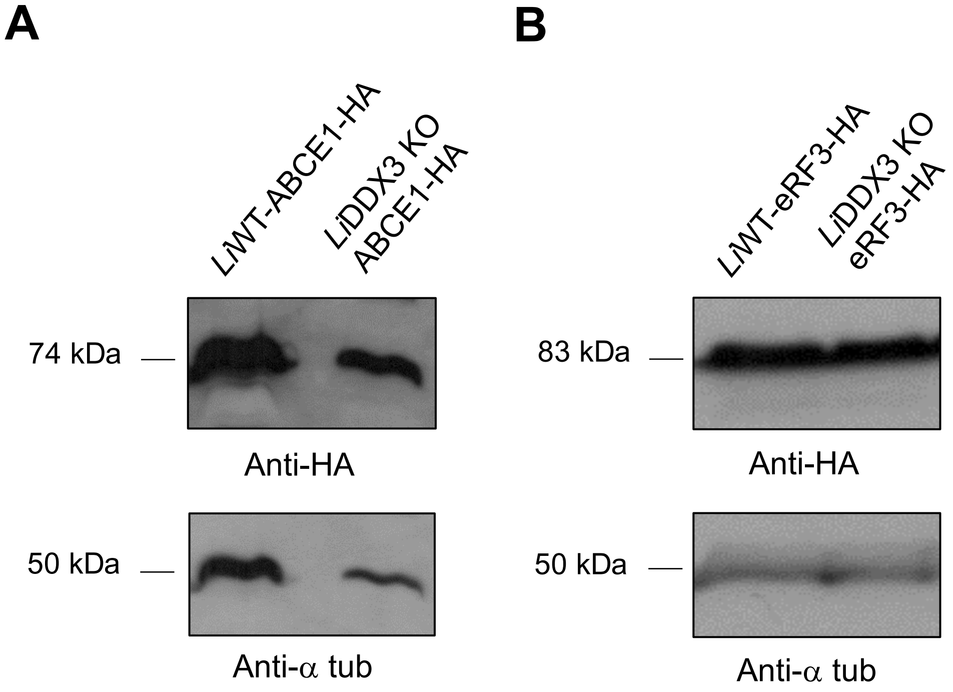
A
B
LiWT-ABCE1-HA
LiDDX3 KO ABCE1-HA
74 kDa
Anti-HA
50 kDa
Anti-α tub
LiWT-eRF3-HA
LiDDX3 KO eRF3-HA
83 kDa
Anti-HA
50 kDa
Anti-α tub

Fig. S7

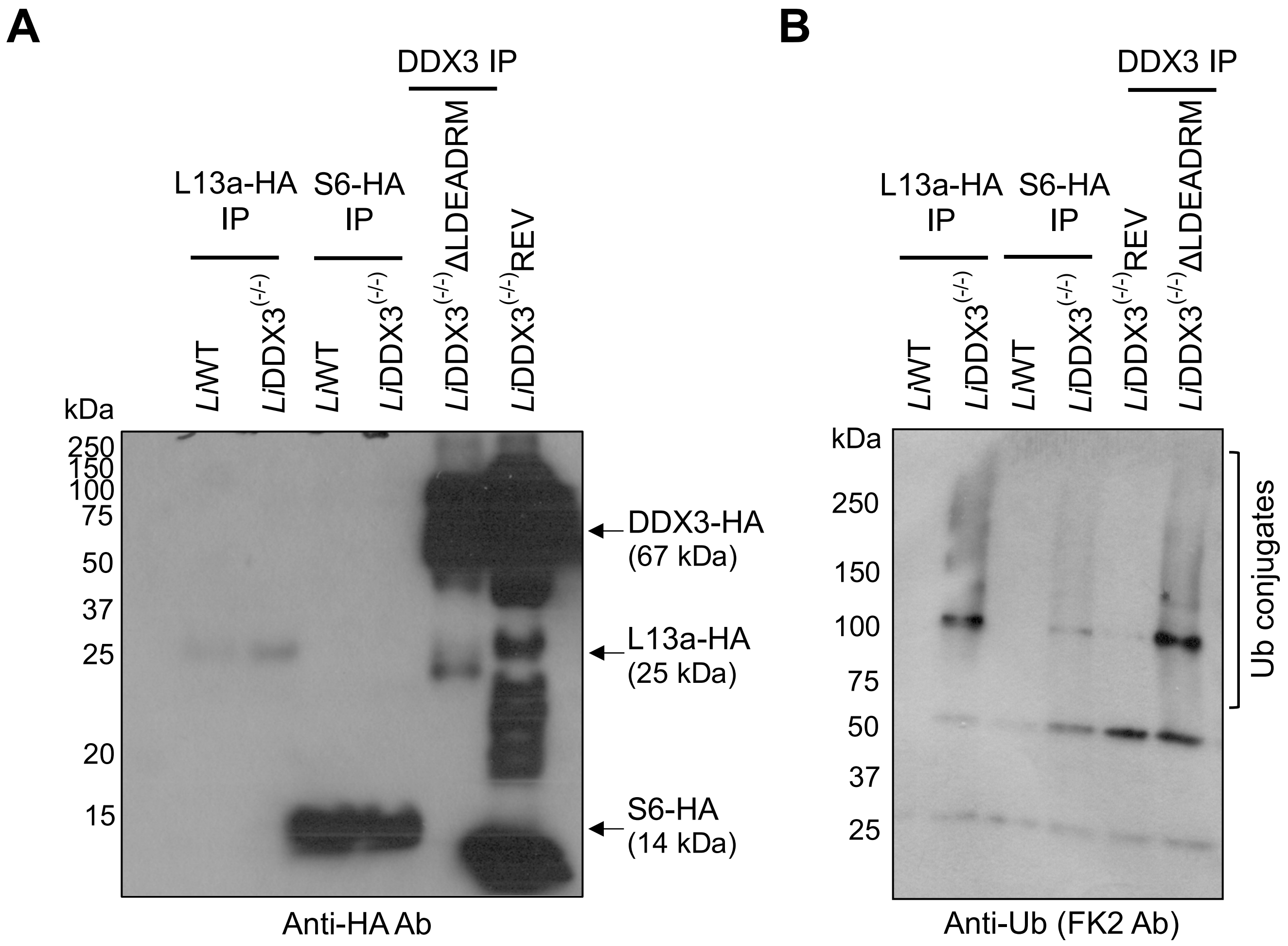
A
DDX3 IP
L13a-HA IP
S6-HA IP
LiWT
LiDDX3(-/-)
LiWT
LiDDX3(-/-)
LiDDX3(-/-)ΔLDEADRM
LiDDX3(-/-)REV
kDa
250
150
100
75
50
37
25
20
15
DDX3-HA (67 kDa)
L13a-HA (25 kDa)
S6-HA (14 kDa)
Anti-HA Ab
B
DDX3 IP
L13a-HA IP
S6-HA IP
LiWT
LiDDX3(-/-)
LiWT
LiDDX3(-/-)
LiDDX3(-/-)REV
LiDDX3(-/-)ΔLDEADRM
kDa
250
150
100
75
50
37
25
Ub conjugates
Anti-Ub (FK2 Ab)

Fig. S8

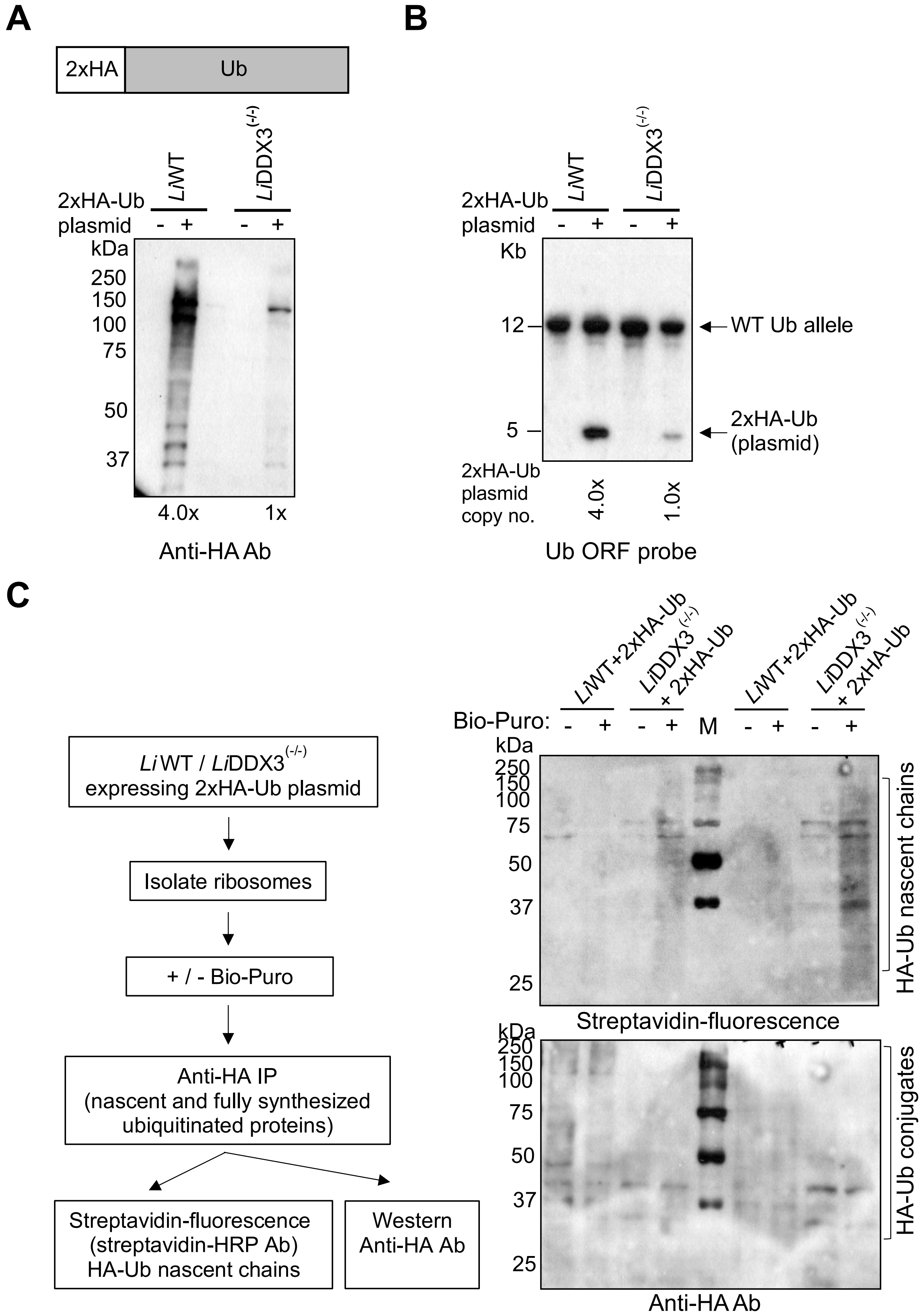
A
2xHA
Ub
2xHA-Ub plasmid
LiWT
LiDDX3(-/-)
kDa
250
150
100
75
50
37
4.0x
1x
Anti-HA Ab
B
Kb
12
5
WT Ub allele
2xHA-Ub (plasmid)
2xHA-Ub plasmid copy no.
4.0x
1.0x
Ub ORF probe
C
LiWT / LiDDX3(-/-) expressing 2xHA-Ub plasmid
Isolate ribosomes
+ / - Bio-Puro
Anti-HA IP (nascent and fully synthesized ubiquitinated proteins)
Streptavidin-fluorescence (streptavidin-HRP Ab) HA-Ub nascent chains
Western Anti-HA Ab
LiWT+2xHA-Ub
LiDDX3(-/-) + 2xHA-Ub
Bio-Puro:
M
HA-Ub nascent chains
Streptavidin-fluorescence
HA-Ub conjugates
Anti-HA Ab

Fig. S9

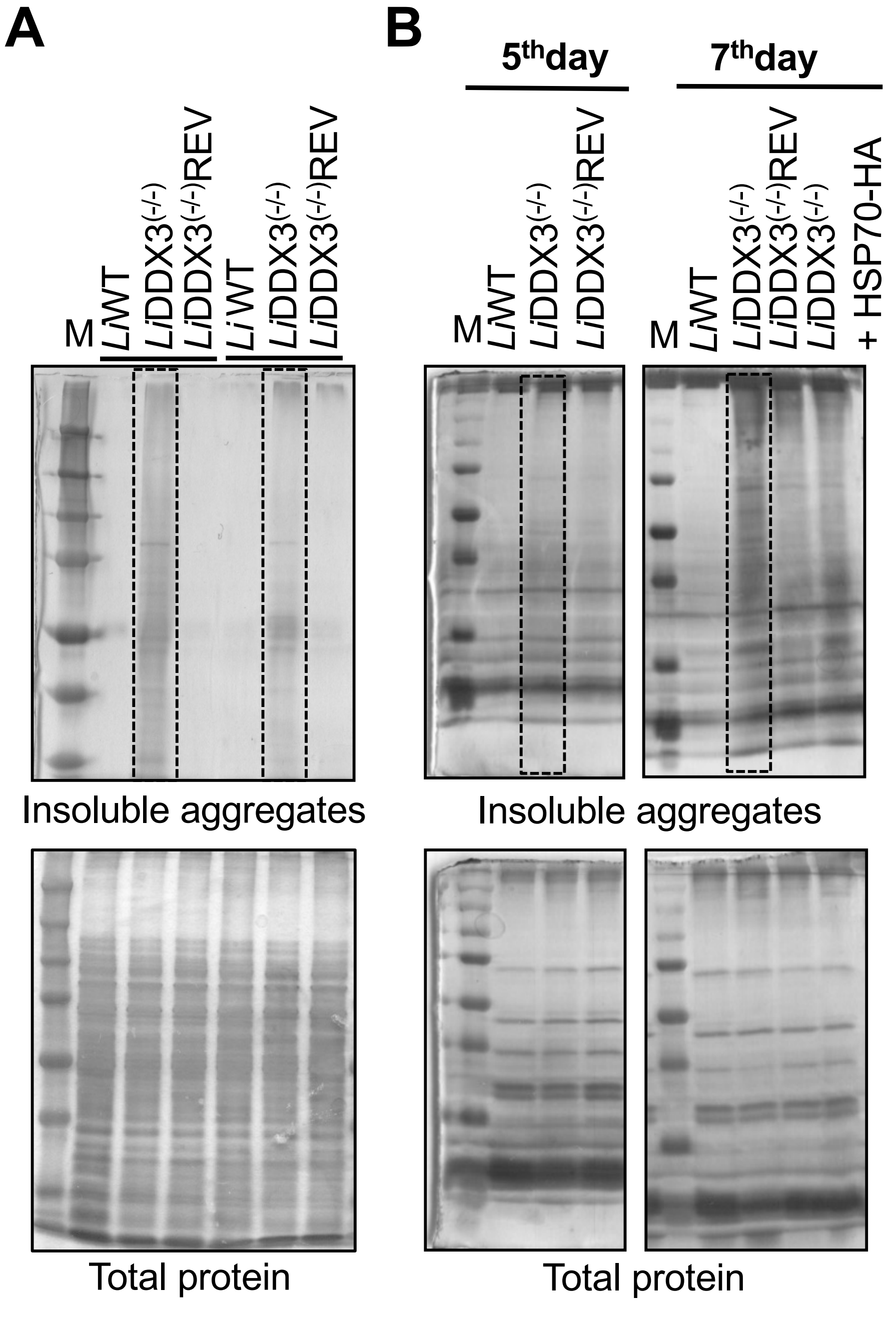
A
B
5thday
7thday
M
LiWT
LiDDX3(-/-)
LiDDX3(-/-)REV
LiWT
LiDDX3(-/-)
LiDDX3(-/-)REV
M
LiWT
LiDDX3(-/-)
LiDDX3(-/-)REV
M
LiWT
LiDDX3(-/-)
LiDDX3(-/-)REV
LiDDX3(-/-)
+ HSP70-HA
Insoluble aggregates
Insoluble aggregates
Total protein
Total protein

Fig. S10

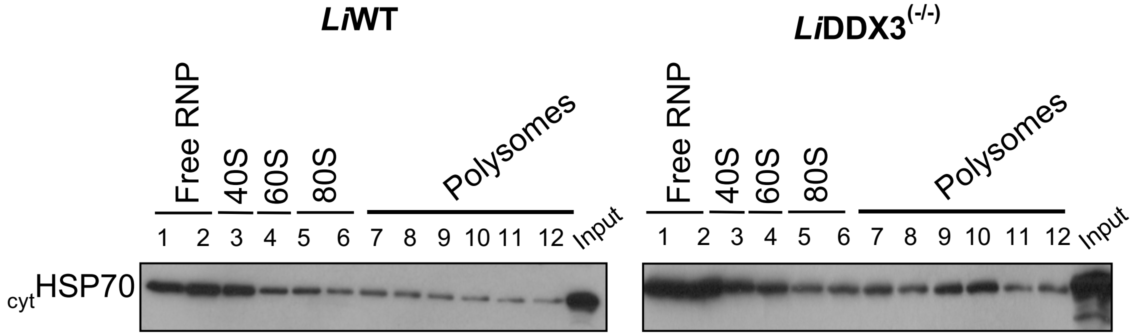

*Li*WT
*Li*DDX3(-/-)
Free RNP
40S
60S
80S
Polysomes
1 2 3 4 5 6 7 8 9 10 11 12 Input
cytHSP70

Fig. S11

Supplementary Tables

Supplementary Table S1. Primers used in this study.

Supplementary Table S2. LC-MS/MS analysis of DDX3 and DDX3ΔLDEADRM pull-downs from *L. infantum* DDX3 knockout (KO) cells ectopically expressing a wild type DDX3 protein or a mutant protein lacking the DEAD-box motif (DDX3ΔLDEADRM), both HA epitope-tagged at their C-terminus.

Supplementary Table S3. Selected proteins identified by label-free quantitative (LFQ) analysis of the total proteome from *L. infantum* wild type (WT) and DDX3 knockout (KO) strains.

Supplementary Table S4. Full list of proteins identified by label-free quantitative (LFQ) analysis of the total proteome from *L. infantum* wild type (WT) and DDX3 knockout (KO) strains.

Supplementary Table S5. Selected proteins identified by label-free quantitative (LFQ) proteomic analysis of ribosomes collected by ultracentrifugation through 35% sucrose cushion from *L. infantum* wild type (WT) and DDX3 knockout (KO) strains.

Supplementary Table S6. Full list of proteins identified by label-free quantitative (LFQ) proteomic analysis of ribosomes collected by ultracentrifugation through 35% sucrose cushion from *L. infantum* wild type (WT) and DDX3 knockout (KO) strains.

Supplementary Table S7. LC-MS/MS analysis of ABCE1-HA immunoprecipitation from *L. infantum* wild type (WT) cells ectopically expressing the ABCE1-HA protein using anti-HA antibody.

Supplementary Table S8. LC-MS/MS analysis of L13a and S6 ribosomal protein pull-downs from *L. infantum* wild type (WT) and DDX3 knockout (KO) strains expressing HA-tagged L13a or S6 ribosomal proteins using anti-HA antibody.
